# Biocatalytic upgrading of fusel oil from bioethanol production to levulinate esters as renewable fuel oxygenates

**DOI:** 10.1186/s13068-026-02816-9

**Published:** 2026-09-08

**Authors:** Yuchen Luo, Mohamed Ismail, Rajni Hatti-Kaul, Sang-Hyun Pyo

**Affiliations:** Biotechnology and Applied Microbiology, Department of Process and Life Science Engineering, Faculty of Engineering, Lund University, 22100 Lund, Sweden

**Keywords:** Biobased fuel additive, Fusel oil, Fusel levulinate, Biocatalytic esterification, Waste-to-value strategy, Sustainable and circular economy

## Abstract

**Background:**

The transition toward a sustainable bioeconomy requires efficient strategies for converting renewable biomass and industrial side streams into value-added chemicals and fuels. Fusel oil is an important coproduct of bioethanol fermentation and levulinic acid (LA), a key platform molecule derived from lignocellulosic biomass, can be upgraded into alkyl levulinates, which are promising green solvents, specialty chemicals, and fuel-related applications.

**Results:**

In this study, a solvent-free enzymatic approach was developed for the synthesis of alkyl levulinates via lipase-catalyzed esterification of levulinic acid with bioethanol-derived fusel oil and its major alcohol components, including 2-methyl-1-butanol and 3-methyl-1-butanol, using immobilized *Candida antarctica* lipase B (Novozym® 435). Under optimized conditions (50 °C, LA:alcohol molar ratio 1:10, enzyme loading 10 wt% and molecular sieve addition equal to substrate mass), quantitative levulinic acid conversion and levulinate yield (~ 98–99%) were achieved within 5 h. In comparison, the secondary alcohol 2-butanol, evaluated for mechanistic comparison, reached a lower conversion (~ 70%) under otherwise identical conditions, consistent with its greater steric hindrance within the enzyme active site. Molecular docking simulations supported experimental observations by elucidating substrate–enzyme interactions and differences in binding affinity. The immobilized enzyme exhibited excellent operational stability, maintaining over 97% LA conversion over five consecutive cycles, and successful operation at 100 mL in a bioreactor highlighted the potential for further scale-up. The solvent-free system reduces process complexity, facilitates downstream processing and enhances process sustainability.

**Conclusion:**

Overall, this work demonstrates an efficient strategy for valorizing fusel oil side stream into high-value levulinate esters, contributing to circular bioeconomy approaches and the development of renewable fuel oxygenates and chemical platforms.

**Graphical abstract:**

**Figure:**
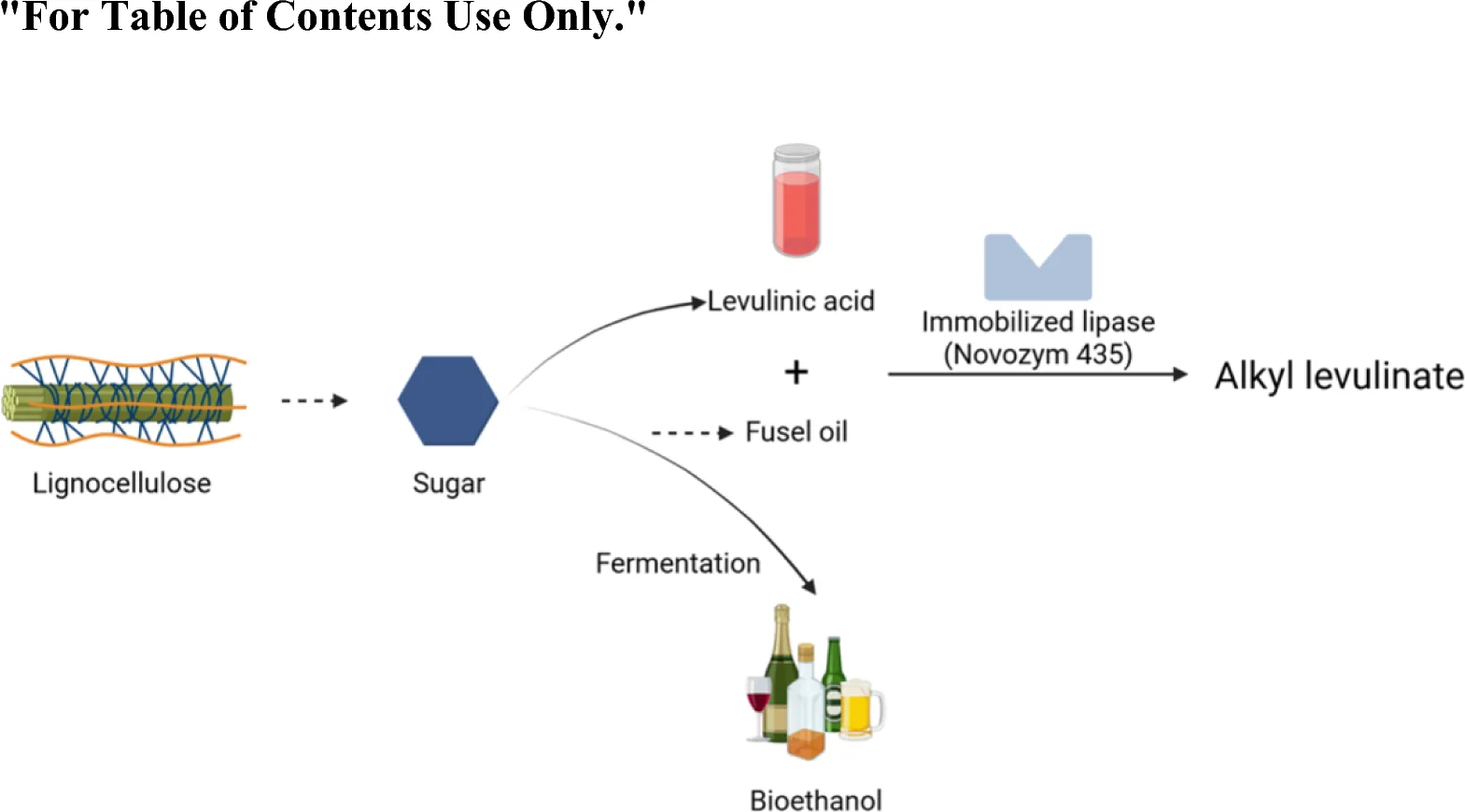

**Supplementary Information:**

The online version contains supplementary material available at https://doi.org/10.1186/s13068-026-02816-9.

## Background

The transition from a fossil-based economy toward a sustainable bioeconomy has attracted increasing global attention due to the environmental impact associated with petroleum-derived resources. Biomass provides an alternative renewable carbon source for the sustainable production of fuels, chemicals, and materials, contributing to efficient use of resources, reduced greenhouse gas emissions, and mitigation of climate change [1, 2]. Advances in biorefinery technologies have enabled the integration of chemical, biological, and catalytic processes to efficiently convert biomass into value-added products such as biofuels, platform chemicals, and materials. These developments play a key role in supporting sustainable development and enhancing energy security, as emphasized by the United Nations Sustainable Development Goals (SDGs) [1, 2].

A key principle of the biobased economy is the valorization of side streams and waste materials through “waste-to-value” strategies, which improve resource efficiency and economic viability [3, 4]. In this context, bioethanol production represents an important industrial platform. While ethanol itself is widely used as a renewable fuel and is also of interest as a building block for chemicals and polymers, its production generates co-products such as dried distillers’ grains with solubles (DDGS), yeast residues, fusel oil, gluten, and carbon dioxide, the valorization of which is crucial for economic viability of the biorefineries [4].

Fusel oil is an important co-product of bioethanol fermentation, produced by yeast metabolism of amino acids via Ehrlich pathway [5]. It is a mixture of volatile, higher alcohols—3-methyl-1-butanol (isoamyl alcohol) and 2-methyl-1-butanol (active amyl alcohol) as the major components (60–70%) besides 15–20% iso-butanol, and trace amounts of n-butanol and n-propanol [6, 7]. Annual production of fusel oil at a leading U.S. ethanol facility (capacity ~1.6 GL year⁻^1^) has been reported at approximately 4 million gallons (15.14 million liters), with potential increases up to 16 million gallons (60.57 ML) following improvements in distillation and offloading capacity [8]. More broadly, considering typical fusel oil formation rates of approximately 1–11 L per 1000 L of ethanol produced, global ethanol production could generate well over 100 million liters of fusel oil annually [9]. Fusel oil components are currently underutilized despite their potential as renewable feedstocks. 3-methyl-1-butanol derived from fusel oil is used in fragrances, cosmetics, and flavors (in the form of esters), while the fossil-based alcohol is used in industrial applications, solvents and plasticizers [10]. By coupling renewable intermediates such as levulinic acid with fusel alcohols from bioethanol production, it becomes possible to develop sustainable pathways toward bio-based esters with applications as solvents, chemical intermediates, and renewable fuel oxygenates. Valorizing this alcohol-rich co-product in this way exemplifies a circular bioeconomy approach, in which a side stream that would otherwise be underutilized, or simply combusted for energy recovery, is instead redirected into a high-value chemical product, improving the overall carbon and economic efficiency of the bioethanol production chain [11–13].

Levulinic acid (LA) is a key bio-based platform molecule produced through the acid-catalyzed dehydration of cellulose-derived sugars such as glucose and fructose, originating from agricultural and forestry biomass [11, 12]. Due to its versatile chemical functionality and renewable origin, levulinic acid has been identified by the United States Department of Energy as one of the top twelve bio-based building blocks for the synthesis of value-added chemicals, liquid fuels, and specialty materials [12]. Among the various derivatives of levulinic acid, alkyl levulinates formed via esterification with alcohols have attracted significant attention because of their wide range of applications as green solvents, fuel additives, and chemical intermediates [12]. As fuel additives, they have been shown to possess characteristics such as low toxicity, high stability and high lubricity and are experiencing high market growth (a compound annual growth rate of 8.8% in the forecast period 2020–2030). These esters also serve as important precursors for the synthesis of γ-valerolactone and other high-value compounds [14, 15]. In addition, alkyl levulinates possess desirable physicochemical properties, including low toxicity, high lubricity, and favorable combustion characteristics, making them promising candidates for biodiesel and renewable fuel oxygenates and formulations [12, 16]. Conventionally, alkyl levulinates can be produced by esterification of levulinic acid with alcohols using homogeneous inorganic acid catalysts [16–18], which although being effective, present challenges related to catalyst recovery, product purification, corrosion, and environmental impact. Heterogeneous acid catalysts, including clay minerals such as kaolinite and montmorillonite, have therefore been explored as alternative proton-donating catalysts that facilitate easier separation and improved sustainability. Recently, microwave-assisted synthesis of the green diesel additive butyl levulinate (BL) has been investigated using a series of tungsten-substituted Keggin-type phosphomolybdic acid catalysts (H₃PMo₁₂₋ₙWₙO₄₀, n = 1–3) [16].

Besides chemical catalysis, enzyme-catalyzed esterification has emerged as an attractive alternative [19, 20]. Lipase-catalyzed reactions offer several advantages, including high selectivity, mild reaction conditions, and reduced formation of side products [21]. The immobilized enzymes further combine the benefits of biocatalysis with heterogeneous catalysis, enabling catalyst recovery, reuse, and simplified downstream processing [20, 21]. Despite the potential of biocatalysis, many reported studies show relatively moderate conversion efficiencies for alkyl levulinate synthesis, particularly when longer-chain alcohols are used. For example, esterification with 1-pentanol (amyl alcohol) typically achieves maximum conversions of approximately 70%, even under excess alcohol conditions, which is rather low for specialty and bulk chemicals considering the subsequent separation costs [22]. Similarly, when 3-methyl-1-butanol is used, conversion of levulinic acid has been reported to reach only about 50% at an alcohol-to-acid molar ratio of 2:1 in organic solvents [10]. These limitations highlight challenges related to resource efficiency, substrate reactivity, equilibrium constraints, and downstream separation.

**Scheme 1.**
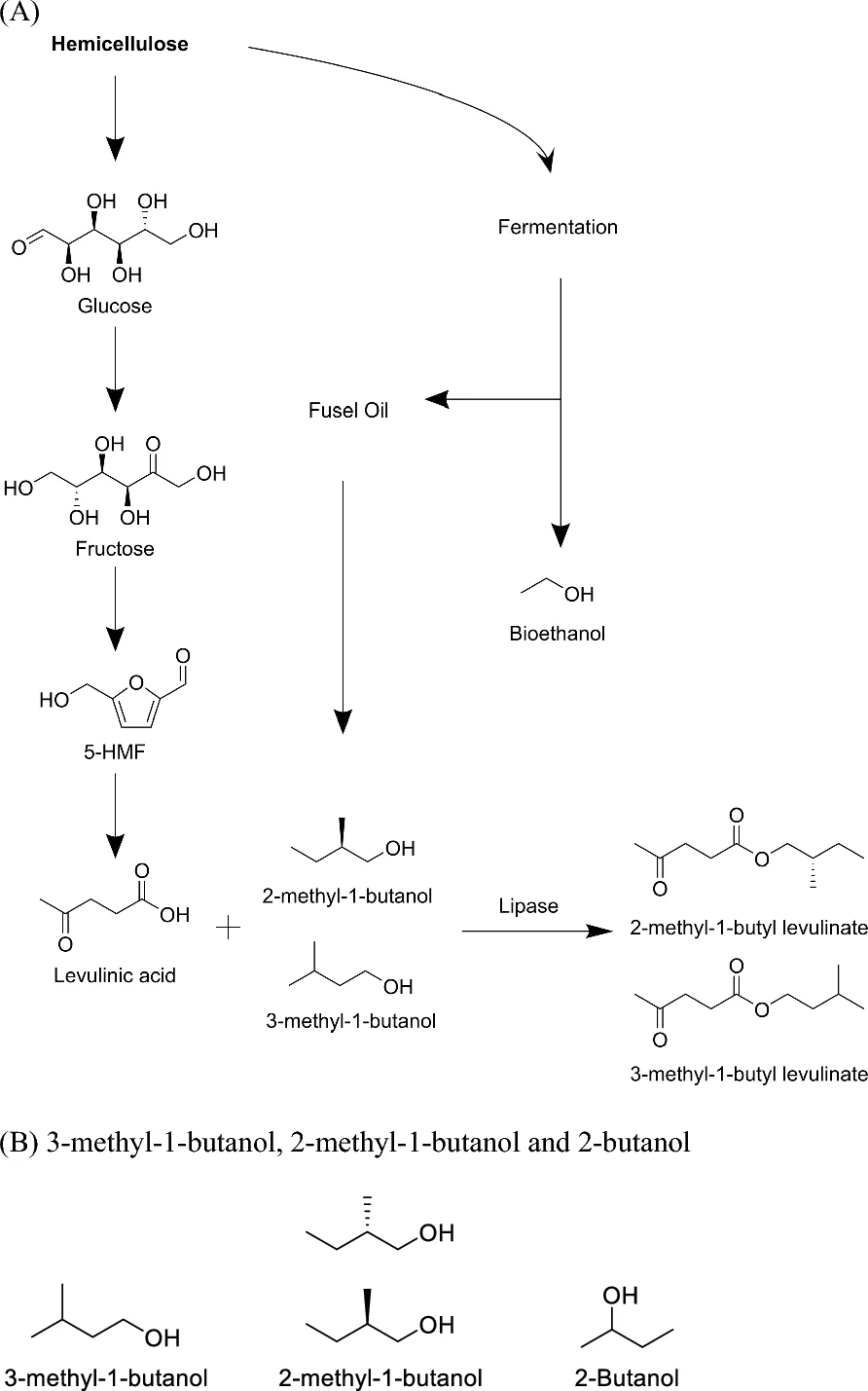
**A** Synthesis of alkyl levulinate from levulinic acid and fusel oil. **B** Alcohols used in this study: 3-methyl-1-butanol, 2-methyl-1-butanol and 2-butanol.

The overall objective of this study is to develop a clean and cost-effective process for producing alkyl levulinates via lipase-catalyzed esterification and to assess the feasibility of valorizing fusel oil from bioethanol production into renewable fuel oxygenates (Scheme 1). Novozym® 435, the immobilized *Candida antarctica* lipase B (CALB) was used as the biocatalyst (Figure S1). Initially, reaction conditions were systematically optimized using 2-methyl-1-butanol as the acyl acceptor, and subsequently the effect of alcohol structure on esterification performance was examined by comparing the reaction with 3-methyl-1-butanol, and 2-butanol, respectively. To gain mechanistic insights into the observed differences in reaction performance, enzyme–substrate interactions were further analyzed through molecular docking simulations. Building on these results, the synthesis of fusel levulinates was evaluated using crude fusel oil obtained from the commercial bioethanol production facility at Lantmännen Biorefinery in Norrköping, Sweden.

## Materials and methods

### Materials

Novozym^®^435 (N435) with a reported activity of 10,000 PLU g⁻^1^ (propyl laurate units per gram) [23, 24] was a generous gift from Novozymes A/S (presently Novonesis), Bagsvaerd, Denmark). Levulinic acid, 2-methyl-1-butanol (2-MB), 3-methyl-1-butanol (3-MB), 2-butanol, molecular sieves (4 Å), and phenolphthalein were purchased from Sigma-Aldrich (USA). Sodium hydroxide was obtained from VWR Chemicals, and ethanol was supplied by SOLVECO (Sweden). Fusel oil was provided by Lantmännen Biorefineries (Norrköping, Sweden). Prior to use, it was distilled and fractionated to remove residual water by rotary evaporation at 70 °C under reduced pressure.

### Novozym^®^435 -catalyzed esterification of levulinic acid with alkyl alcohols

The lipase-catalyzed esterification of levulinic acid (LA) with alkyl alcohols was carried out under controlled reaction conditions to evaluate the effects of biocatalyst loading, reaction temperature, substrate molar ratio, molecular sieve addition, enzyme recyclability, and scale-up performance. Esterification reactions were conducted in 5-mL glass vials. Reaction mixtures were incubated at controlled temperature using a heating thermomixer (MHR 13, Hettich Benelux, Netherlands) with agitation at 600 rpm. During the reaction, aliquots (20–30 μL) were periodically withdrawn for analyses.

*Effect of biocatalyst loading:* the influence of enzyme loading on ester formation was investigated under fixed reaction conditions (50 °C, LA: 2-methyl-1-butanol (2-MB) molar ratio of 1:10, and molecular sieve loading equal to the mass of LA). Novozym^®^435 was added at 5%, 10%, 15%, and 20% (w/w) relative to the acid substrate. Since the manufacturer-reported specific activity of 10,000 PLU g⁻^1^ for N435 does not necessarily reflect catalytic activity toward levulinic acid esterification, the biocatalyst dosage in this study is expressed on a mass basis (% w/w relative to LA), consistent with common practice in the esterification literature [23, 24].

*Effect of reaction temperature*: using optimized biocatalyst loading (10% w/w relative to LA), an LA:2-MB molar ratio of 1:10, and molecular sieves equal in mass to LA, the catalytic rate and final conversion were evaluated at reaction temperatures of 40, 50, and 60 °C.

*Effect of substrate molar ratio*: the effect of alcohol excess was studied by varying the LA: 2-MB molar ratio from 1:1 to 1:10 under otherwise constant conditions (50 °C, biocatalyst loading of 10% w/w relative to LA, and molecular sieve mass equal to LA).

*Effect of molecular sieves*: the effect of varying the molecular sieve loading at 0%, 50%, and 100% (w/w relative to LA) for water removal was investigated at 10% w/w enzyme loading relative to LA, LA: 2-MB molar ratio of 1:10, and temperature 50 °C).

The reaction samples were periodically withdrawn in all the experiments for analysis as described in Sect. 2.5.

### Reusability test of Novozym^®^435 for esterification of levulinic acid with 2-methyl-1-butanol

Enzyme reusability was evaluated in 1–2 mL reaction volumes under optimized conditions (LA:2-MB molar ratio 1:10, enzyme loading 10% w/w relative to LA, molecular sieve loading 100% w/w relative to LA, temperature 50 °C, agitation speed 600 rpm, and reaction time 240 min). After each 4 h reaction cycle, the immobilized enzyme was recovered by filtration, washed 2–3 times with 1 mL of 2-methyl-1-butanol to remove residual reactants and products, and reused in fresh reaction mixtures. Catalytic performance and stability were assessed by monitoring reaction rates and levulinic acid conversion over successive cycles.

### Scaling-up lipase-catalyzed esterification of levulinic acid with 3-methyl-1-butanol and fusel alcohol

Scale-up experiments were conducted in 100 mL volume in a 200-mL working-volume bioreactor equipped with a SpinChem® RBR S2 system (Spinchem, Umeå, Sweden). Reaction conditions were maintained at 50 °C with an LA:alcohol at molar ratio of 1:10, enzyme loading of 10% (w/w relative to LA), molecular sieve loading of 100% (w/w relative to LA), and agitation speed was set to 400 rpm. 3-Methyl-1-butanol and fusel alcohol were employed in the biocatalytic esterification, respectively. The samples (1 mL) were withdrawn at 15, 30, 60, 90, 120, 180, 240, and 300 min using a long-tip pipette. After completion of the esterification reaction, excess alcohol was removed by rotary evaporation at 95 °C under reduced pressure (20 mbar) and a rotation speed of 150 rpm for 30–60 min.

### Analytical procedures and calculations

Quantitative analysis of levulinic acid, alkyl levulinates and reaction intermediates was performed using colorimetric titration, high-performance liquid chromatography (HPLC), and nuclear magnetic resonance spectroscopy (^1^H- and ^13^C-NMR).

During the reaction, aliquots were periodically withdrawn and diluted in 70% (v/v) aqueous ethanol prior to titration. Levulinic acid conversion was determined by titration with NaOH solution (20 mmol L⁻^1^), using phenolphthalein (50 μL) as the endpoint indicator. All experiments were conducted in duplicate. Acid conversion (%) and initial reaction rate (µmol/min) were calculated according to Eqs. (1), (2), and (3).

1 $$Conversion\left(\%\right)=1-\frac{{n}_{NaOH,sample}-{n}_{NaOH,blank}}{{n}_{LA,0}}\times 100\%$$

2 $${n}_{NaOH}={C}_{NaOH}\times ({V}_{sample}-{V}_{blank})$$

3 $${v}_{0}(umol/min)={slope}_{t\in [\mathrm{0,60}min]}({n}_{Consumption }vs t)$$

HPLC analyses of levulinic acid were performed using a fast acid analysis column coupled with a guard column (Bio-Rad, Richmond, CA, USA). The column temperature was maintained at 65 °C using a column oven (Shimadzu, Tokyo, Japan). Samples were diluted with Milli-Q water, acidified with 10% (v/v) sulfuric acid (25 μL mL⁻^1^ sample), and filtered through a 0.45 μm membrane prior to analysis. A sample volume of 40 μL was injected, and separation was achieved using 10 mM H₂SO₄ as the mobile phase at a flow rate of 0.6 mL min⁻^1^. External standards were used for compound identification and quantification.

Substrates, intermediates, and purified alkyl levulinates were characterized by FTIR and NMR spectroscopy. ^1^H- and ^13^C-NMR spectra were recorded on a Bruker UltraShield Plus 400 MHz spectrometer (Germany) using CDCl₃ as solvent. Chemical shifts (δ) are reported in ppm (Figs. S2–S4). FTIR spectra were obtained using a Nicolet iS5 spectrometer (Thermo Scientific, USA) over the range 500–4000 cm⁻^1^ (Figure S2-S4). The fusel oil sample was prepared by removing approximately 30% of the sample volume using a vacuum rotary evaporator to eliminate water and small amounts of lower alcohols, and by using anhydrous sodium sulphate as a desiccant to remove any remaining water from the residual sample.

Analysis of alkyl levulinates, alcohols, and fusel oil was performed by gas chromatography–mass spectrometry (GC–MS) using a Hewlett-Packard HP 6890 GC coupled with a Hewlett-Packard 5973 mass selective detector (Figure S7). GC–MS analysis was performed using an HP-5MS capillary column (30 m × 0.250 mm; Agilent Technologies, USA). The oven temperature was increased from 40 to 250 °C at a heating rate of 20 °C/min. Samples diluted in acetonitrile to a concentration of 0.1–2 mg/mL were injected in split mode at an injector temperature of 275 °C.

### Molecular docking and MD simulation

To investigate substrate binding and catalytic positioning, molecular docking combined with molecular dynamics (MD) simulations was performed using *C. antarctica* lipase B (CALB) as the model enzyme [25, 26]. Lipase-catalyzed esterification reactions are known to proceed via a Ping–Pong Bi–Bi mechanism, involving the formation of a covalent acyl–enzyme intermediate [27]; hence a covalent acyl–enzyme intermediate was constructed by linking levulinic acid to the catalytic residue Ser105, representing the acylated state of the enzyme. This acyl–enzyme complex was used as the receptor for docking of alcohol substrates, including 2-methyl-1-butanol, 3-methyl-1-butanol, and 2-butanol. In practical systems, alcohols are present in excess and serve as reaction medium. High alcohol concentrations may influence enzyme activity by altering water activity, disrupting essential hydration layers required for enzyme flexibility and function [28–30], or inducing nonspecific interactions with the enzyme surface [31–33]. Thus, prior to docking, the acyl–enzyme complex was subjected to a 3 ns MD simulation to allow structural relaxation and stabilization of the active site. Representative snapshots were extracted along the trajectory and used for subsequent docking analyses to account for enzyme conformational flexibility.

Molecular docking was performed using the YASARA software, and binding energy (kcal/mol; higher values indicate stronger predicted binding) for each docked pose was used to estimate relative binding affinity and predicted dissociation constants (K_d_, pM). The latter was derived via the standard thermodynamic relationship between free energy of binding and equilibrium constant (ΔG = − RT ln K_d_) at 298 K. For each alcohol, binding energy and K_d_ were averaged across the three representative MD-derived snapshots to obtain a mean ± standard deviation, and the coefficient of variation (CV = SD/mean × 100%) was used to quantify snapshot-to-snapshot variability (Table 1). Pearson correlation coefficients (r) were calculated between each alcohol's mean binding energy, and separately a mean dissociation constant and its experimentally measured initial reaction rate.

**Table 1:** Molecular docking characteristics and experimental reaction rates for different alcohol substrates (2-methyl-1-butanol, 3-methyl-1-butanol, and 2-butanol) with *C. antarctica* lipase

Alcohol	Binding energy (kcal/mol)	Dissoc. Const (pM)	Initial reaction rate (μmol/min)
3-Methyl-1-butanol	3.493	2,751,674,368	0.15
	3.825	1,571,223,040	
	3.919	1,340,710,144	
2-Methyl-1-butanol	3.559	2,461,605,376	0.20
	3.793	1,658,418,560	
	3.891	1,405,591,680	
2-Butanol	2.739	9,823,954,944	0.08
	3.438	3,019,343,872	
	2.905	7,423,466,496	

## Results and discussion

Lipase-catalyzed esterification is undoubtedly among the most extensively studied biocatalytic processes reported in the literature [34], and some processes have been successfully demonstrated at larger scale [35, 36]. Several studies have reported the enzymatic synthesis of alkyl levulinates, with ester yields strongly influenced by catalyst type, reaction medium, substrate ratio, and operating conditions. Consequently, significant variations in conversion efficiency and reaction time have been reported across the literature (Table 1). N435 is a biocatalyst comprising *Candida antarctica* lipase B adsorbed by interfacial action to Lewatit VP OC 1600, a macroporous acrylic polymer resin with interconnected pores, at a loading of 10 wt% [24]. Fusel oil is a complex mixture of higher alcohols, and its exact composition depends on fermentation substrate and process conditions. Therefore, in this study, 2-methyl-1-butanol, 3-methyl-1-butanol, 2-butanol, and crude fusel oil (dried) were employed and compared for the esterification with levulinic acid using the immobilized lipase, Novozym® 435 under solvent-free conditions.

### Optimization of Novozym® 435 -catalyzed esterification of levulinic acid with 2-methyl-1-butanol

**Fig. 1:**
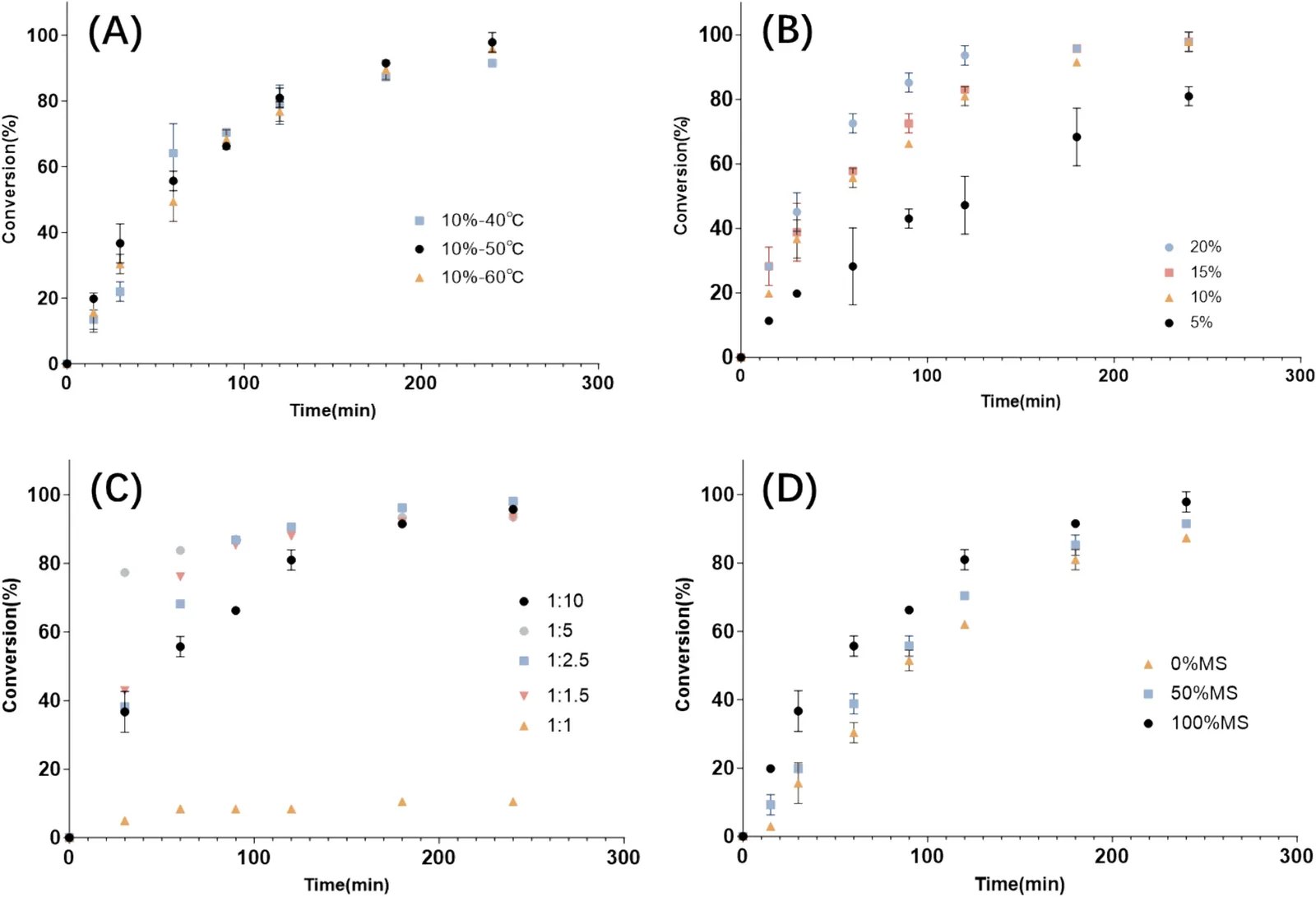
Optimization of Novozym® 435-catalyzed esterification of levulinic acid (LA) with 2-methyl-1-butanol (2-MB) during reaction time of 240 min: **A** Effect of biocatalyst loading in reaction using LA:2-MB molar ratio of 1:10, temperature of 50 °C, agitation speed of 600 rpm, and molecular sieve loading of 100% (w/w relative to LA). **B** Effect of reaction temperature at LA:2-MB molar ratio 1:10, enzyme loading 10% (w/w relative to LA), agitation speed 600 rpm, and molecular sieve loading 100% (w/w relative to LA). **C** Effect of substrate molar ratio (LA:2-MB) at enzyme loading 10% (w/w relative to LA), temperature 50 °C, agitation speed 600 rpm, and molecular sieve loading 100% (w/w relative to LA). **D** Effect of molecular sieve loading at LA:2-MB molar ratio 1:10, enzyme loading 10% (w/w relative to LA), temperature 50 °C, and agitation speed 600 rpm

Since 2-methyl-1-butanol represents a major component of fusel oil, it was used as the acyl acceptor for initial optimization of esterification reactions with levulinic acid. The key reaction parameters, including enzyme loading, temperature, substrate molar ratio, and molecular sieve dosage, were systematically investigated (Fig. 1).

## Effect of biocatalyst amount

Biocatalyst loading is a critical parameter influencing both catalytic performance and process economics [20, 21]. Although increasing enzyme concentration generally enhances reaction rate, excessive loading increases production costs. Therefore, an optimal balance between catalytic efficiency and economic feasibility must be established. Increasing the amount of Novozym® 435 from 5 to 10% (w/w relative to LA) led to a significant increase in reaction rate (Fig. 1B), while at 10% and 15% biocatalyst loading, similar reaction rates were observed, achieving nearly complete conversion (~ 100%) within approximately 4 h. In contrast, 5% biocatalyst loading resulted in only ~80% conversion under the same conditions. Although a slight improvement in reaction rate was observed at 20% loading, the marginal gain did not justify the increased enzyme consumption. Therefore, 10% (w/w relative to LA) was selected for subsequent experiments, providing an optimal balance between catalytic efficiency and cost.

## Effect of reaction temperature

Reaction temperature constitutes one of the key parameters for optimizing reaction efficiency as it influences molecular kinetic energy and can enhance reaction rates while leading to conformational changes at high temperatures and compromising enzyme stability [37]. Novozym® 435 is known to possess remarkably high stability in hydrophobic solvents like diphenyl ether and toluene [38], though its stability can vary significantly based on the specific solvent, water activity and temperature [39]. The substrates in the present reaction are relatively polar; hence for determining the optimal conditions, the reaction temperature was varied between 40 °C and 60 °C. As shown in Fig. 1B, no significant differences in reaction rate or final conversion were observed across this temperature range. Consequently, 50 °C was selected as the standard reaction temperature for subsequent experiments.

## Effect of molar ratio of the substrates

The substrate molar ratio influences both reaction kinetics and equilibrium position [28, 40]. In lipase-catalyzed esterification, alcohol can act both as reactant and reaction medium in solvent-free systems. However, excessive alcohol concentrations may inhibit enzyme activity through two mechanisms—either by inducing conformational changes in lipase that lead to aggregation or by causing the enzyme to detach from the solid carrier [26–28, 39].

To determine the optimal molar ratio, LA:2-MB ratios ranging from 1:1 to 1:10 were investigated (1:1, 1:1.5, 1:2.5, 1:5, and 1:10). As shown in Fig. 1C, all ratios except 1:1 achieved high conversion within 4 h. Ratios of 1:1.5, 1:2.5, and 1:5 exhibited comparable and relatively rapid reaction rates. When the molar ratio was increased to 1:10, a slight decrease in reaction rate was observed, possibly due to alcohol-induced inhibition of lipase activity. At lower alcohol ratios, the proportion of solid materials (the immobilized enzyme and molecular sieves) relative to liquid phase was higher, potentially limiting mass transfer. Considering conversion efficiency, enzyme stability, and system rheology, a molar ratio of 1:10 was selected as the standard condition for subsequent experiments. In contrast to previous studies [10, 40–42] in which solvents such as MTBE or MIBK were employed to achieve high yields, 2-methyl-1-butanol serves both as acyl acceptor and reaction medium in this work. While the addition of solvents can enhance mass transfer, they introduce additional downstream separation steps. For instance, the boiling points of active amyl alcohol (127.5 °C) and MIBK (117 °C) are relatively close, complicating separation and solvent recovery at larger scale. By operating under solvent-free conditions, the ester product can be purified simply by removing excess alcohol, which can be directly reused in subsequent batches.

## Effect of molecular sieves as water scavenger

Efficient water removal is essential to drive the reaction equilibrium toward ester formation and avoid hydrolysis of the product, which can also help to maintain enzyme activity and stability [43, 44]. Use of molecular sieves to adsorb water directly from the reaction mixture is among the effective methods commonly used. As shown in Fig. 1D, increasing the amount of molecular sieves (4 Å) from 0 to 100% (w/w relative to LA) enhanced both reaction rate and final conversion. At 100% (w/w relative to LA), nearly complete conversion (> 98%) was achieved within 4 h, while lower sieve loadings resulted in incomplete conversion. Complete conversion of levulinic acid to ester product is particularly advantageous, as it eliminates the need for separation of unreacted acid from the ester product.

Based on systematic optimization, the optimal reaction conditions were identified as follows: enzyme loading of 10% (w/w relative to LA), molecular sieve loading of 100% (w/w relative to LA), reaction temperature of 50 °C, and an LA:alcohol molar ratio of 1:10. Under these conditions, Novozym® 435-catalyzed esterification achieved near 100% conversion of acid within 5 h, and high selectivity toward 2-methyl-1-butyl levulinate in a solvent-free system, demonstrating the feasibility of efficient enzymatic upgrading of an industrial by-product.

### Effect of alcohol composition in fusel oil on the esterification of levulinic acid

## Effect of different alcohols on esterification performance

**Fig. 2:**
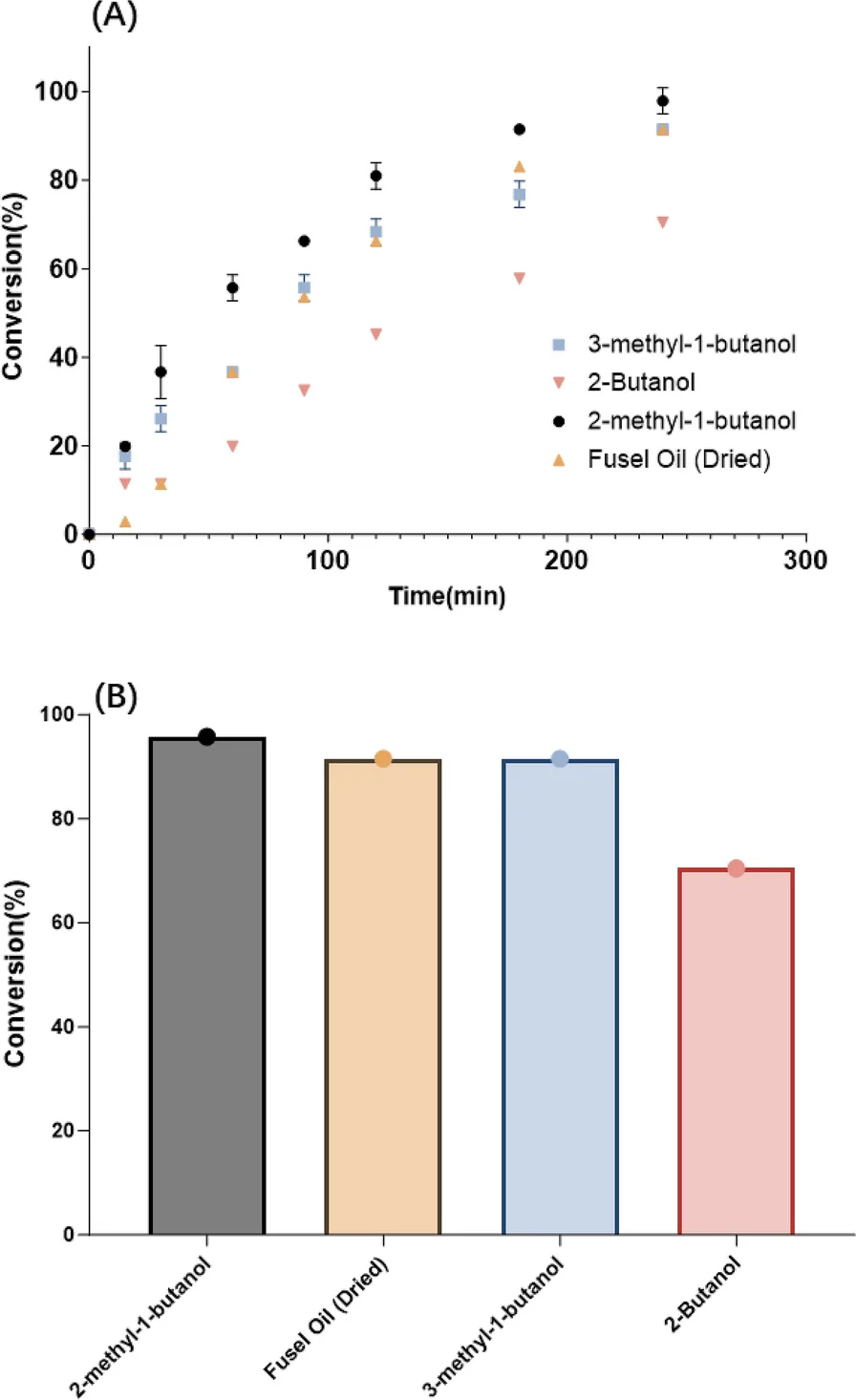
Effect of alcohol type on esterification with levulinic acid (LA) catalyzed by Novozym® 435: **A** Reaction time course, and **B** degree of conversion of LA after 4 h. Reaction conditions: total reaction time 240 min; substrate ratio LA:alcohol = 100 mg:1 mL; biocatalyst loading 10% (w/w relative to LA); agitation speed 600 rpm; temperature 50 °C; and molecular sieve loading 100% (w/w relative to LA)

To evaluate the feasibility of utilizing fusel alcohol mixtures directly, the lipase-catalyzed esterification of levulinic acid was performed also with other alcohol components and dehydrated fusel oil under the optimized conditions established in Sect. 3.1. As shown in Fig. 2, 2-methyl-1-butanol exhibited the highest initial reaction rate (0.2 μmol/min), followed by 3-methyl-1-butanol (0.15 μmol/min), whereas 2-butanol showed the lowest rate (0.08 μmol/min). 3-Methyl-1-butanol and dehydrated fusel oil also achieved near-complete LA conversion within 4–5 h, demonstrating that crude fusel mixtures can be effectively utilized without prior purification of individual alcohol components. In contrast, 2-butanol, which was compared with fusel alcohol, exhibited significantly lower conversion under identical conditions. Being a secondary alcohol, 2-butanol introduces steric hindrance, making it less favorable for fitting in the enzyme active site. Novozym® 435 has structural preference for primary alcohols, which can readily enter the active site and form the acyl-enzyme intermediate [45].

**Fig. 3:**
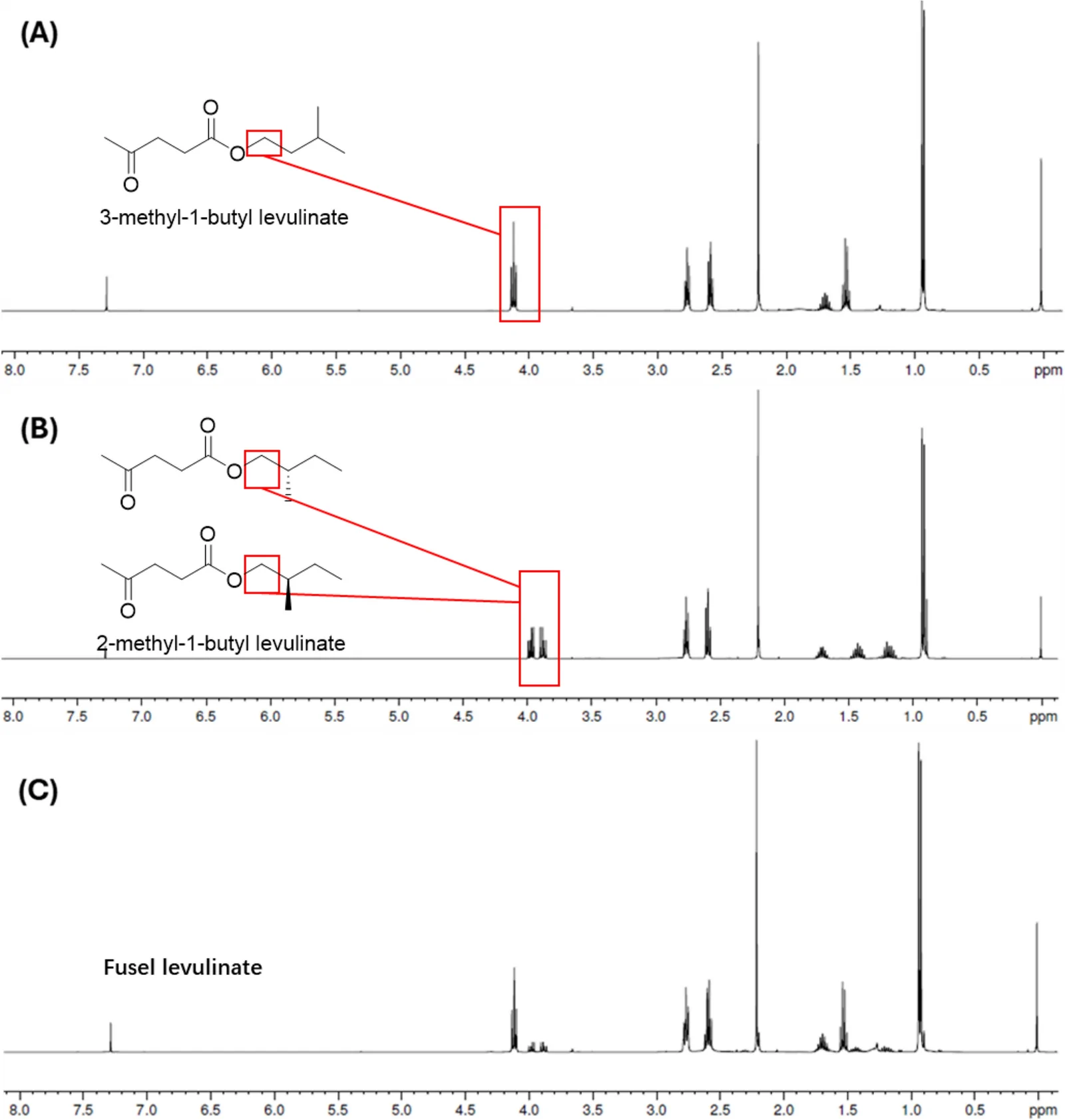
Proton NMR spectra of **A** 3-methyl-1-butyl levulinate, **B** 2-methyl-1-butyl levulinate, and **C** fusel levulinate after removal of water and lower alcohols

For fusel oil derived from bioethanol production, which mainly contains 2-methyl-1-butanol and 3-methyl-1-butanol (Figure S6, S7), the product distribution reflected a similar ratio between the corresponding levulinate esters, although a slight decrease in the proportion of the 2-methyl-1-butanol-derived ester was observed (Fig. 3).

As shown in the ^1^H-NMR spectra in Figs. 3, S4, and S6, the signals in the 3.5–4.0 ppm region serve as characteristic indicators for active amyl alcohol and isoamyl alcohol derivatives. In the ^1^H-NMR spectrum of the fusel oil feed, the peak area ratio corresponding to active amyl alcohol and isoamyl alcohol was approximately 0.41:1. After 5 h of reaction, this ratio decreased to 0.35:1 in the ^1^H-NMR spectrum of the mixed ester products. This indicates that, under identical reaction conditions, ester formation from 2-methyl-1-butanol in fusel oil proceeded slightly more slowly than that from 3-methyl-1-butanol.

The reduced reactivity of 2-butanol can be attributed to its secondary alcohol structure, which introduces greater steric hindrance and reduces accessibility to the acyl-enzyme intermediate [45]. These structural effects are particularly relevant in enzymatic esterification, where geometric alignment within the active site plays a critical role in catalysis.

## Molecular docking analysis of alcohol–enzyme interactions

**Fig. 4:**
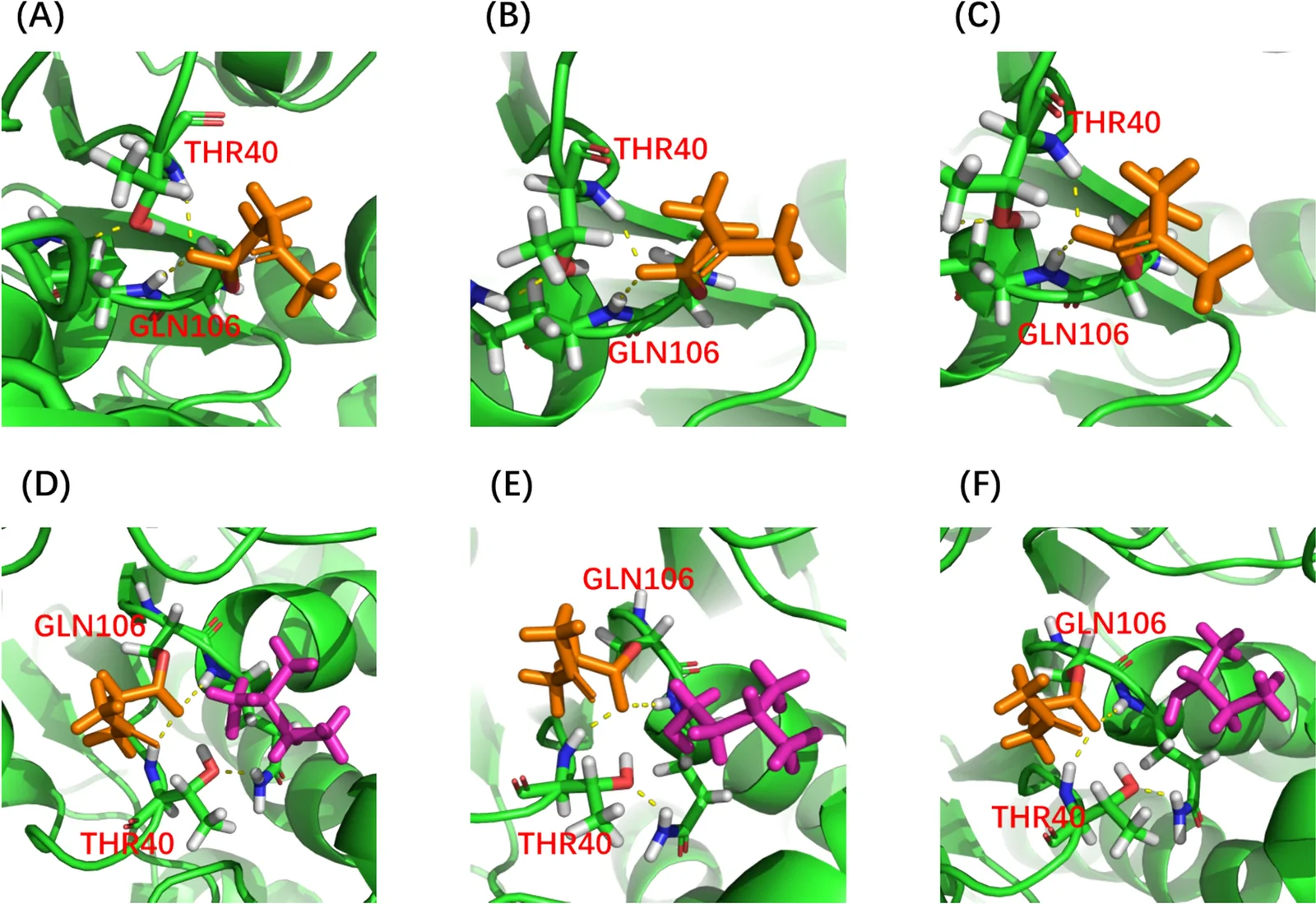
Docking of levulinic acid (LA) on *C. antarctica* lipase B indicating the formation of the productive binding mode within the active site with hydrogen bonding required for the substrate stabilization in the tetrahedral intermediate (THI). Hydrogen bonding between: **A** the carbonyl oxygen and the side-chain hydroxyl hydrogen of Thr40 and the amide hydrogen of Gln106, (**B**) the carbonyl oxygen and the backbone amide hydrogen of Thr40 and the amide hydrogen of Gln106, and **C** the carbonyl oxygen and both the side-chain hydroxyl and backbone amide hydrogens of Thr40. Comparison of docking conformations for **D** 2-methyl-1-butanol, **E** 3-methyl-1-butanol, and **F** 2-butanol

Molecular docking and molecular dynamics simulations were employed to elucidate the interactions between alcohol substrates and the active site of *C. antarctica* lipase B (CALB), particularly focusing on the formation and stabilization of the acyl-enzyme intermediate within the catalytic pocket [46, 47]. Across the selected molecular dynamics snapshots of the acyl-enzyme intermediate within the oxyanion hole at 1.8, 1.9, and 2.0 ns (Fig. 4A–C), the intermediate was consistently stabilized by hydrogen-bonding interactions, primarily involving residues Thr40 and Gln106. This observation is consistent with the established catalytic mechanism of CALB, in which the oxyanion hole plays a critical role in stabilizing high-energy intermediates during catalysis [46].

The docking results showed clear differences in predicted binding strength and catalytic positioning among the investigated alcohols, correlating well with experimentally determined initial reaction rates (Table 1). In the YASARA scoring function, higher binding scores correspond to stronger predicted binding interactions and are consistent with lower predicted dissociation constants. The observed reaction rates are consistent with the docking results, where the two pentanol isomers displayed higher binding scores and lower predicted dissociation constants compared to 2-butanol (mean binding energy ~3.75 kcal/mol for both pentanol isomers vs. 3.03 kcal/mol for 2-butanol; mean dissociation constant ~1.9 × 10^9^ pM for the pentanols vs. 6.8 × 10^9^ pM for 2-butanol, Table 1), indicating stronger and more stable binding within the active site. The similar binding energies for 3-methyl-1-butanol and 2-methyl-1-butanol are consistent with their similarly high reactivity relative to 2-butanol. Likewise, the initial-rate measurements showed that, although 2-methyl-1-butanol gave a slightly higher initial rate, its reactivity remained comparable to that of 3-methyl-1-butanol. In contrast, 2-butanol showed a significantly lower reaction rate (0.08 μmol/min) than the two amyl alcohols (0.15 and 0.2 μmol/min), which is also in agreement with the molecular docking results.

In addition to weaker overall binding, 2-butanol exhibited greater variability in binding scores across different MD snapshots (Table 1) (coefficient of variation of 12% in binding energy vs. 5–6% for the two amyl alcohols), suggesting less consistent accommodation within the active site. This variability indicates a reduced probability of forming catalytically productive conformations required for the deacylation step. The lower reactivity of 2-butanol is therefore best interpreted in terms of reduced compatibility with the productive deacylation geometry in CALB rather than simple steric exclusion from the active site. Although CALB is known to accommodate secondary alcohols through a stereospecificity pocket [21, 48, 49], efficient catalysis requires precise alignment of the alcohol with the acyl-enzyme intermediate and the catalytic His224-mediated proton-transfer network. In this context, the secondary structure of 2-butanol may limit optimal positioning of the hydroxyl group, thereby reducing the frequency of near-attack conformations and lowering catalytic turnover.

Overall, the results indicate that esterification kinetics in CALB are governed not only by substrate binding strength but also by steric accessibility and conformational compatibility within the active site. Higher YASARA binding scores and lower predicted dissociation constants are associated with stronger binding; however, catalytic efficiency is more directly determined by the ability of the substrate to adopt catalytically productive conformations that facilitate the deacylation step.

Across the three alcohols, the mean binding energy correlated positively with the experimentally measured initial reaction rate (Pearson r = 0.91), while the mean dissociation constant correlated negatively (r = − 0.91), supporting a link between predicted binding affinity and catalytic performance. Because only three alcohols were compared, these correlations should be regarded as illustrative rather than statistically robust, and are intended to complement, rather than substitute for, the mechanistic interpretation based on binding geometry and active-site accommodation described above.

### Reusability of Novozym® 435 for esterification of levulinic acid with 2-methyl-1-butanol

**Fig. 5:**
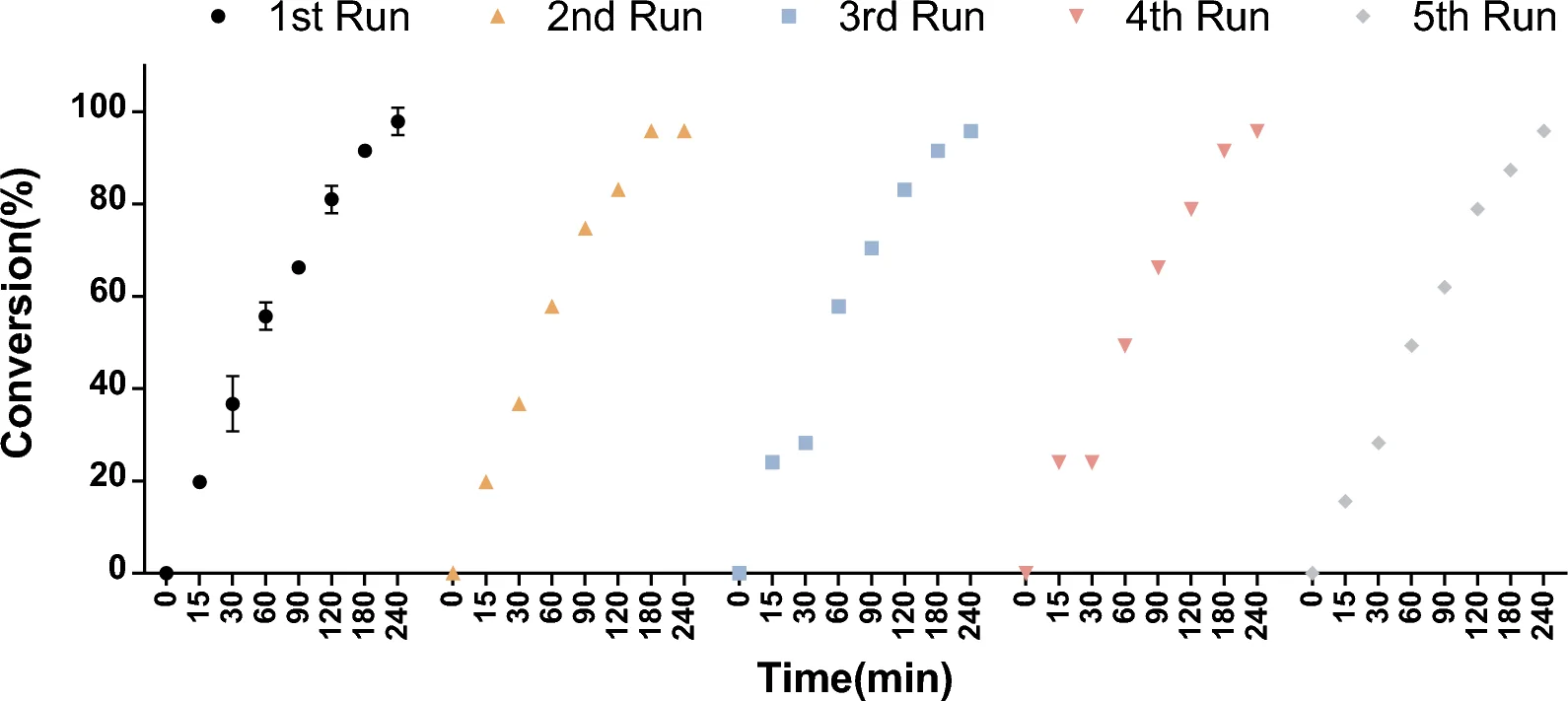
Reusability of Novozym® 435 for esterification of levulinic acid (LA) with 2-methyl-1-butanol (2-MB). Reaction conditions: LA:2-MB molar ratio 1:10, enzyme loading 10% (w/w relative to LA), agitation speed 600 rpm, temperature 50 °C, and molecular sieve loading 100% (w/w relative to LA) over a period of 240 min. After each cycle, the catalyst was recovered by filtration, washed with 2-methyl-1-butanol, and reused in the subsequent reaction

One of the key advantages of immobilized enzymes compared with free enzymes is their potential for reuse, which directly influences process sustainability and economic feasibility. Therefore, the operational stability and reusability of Novozym® 435 were evaluated through five consecutive batch reactions under the optimized conditions established in Sect. 3.1 (Fig. 5).

As shown in Fig. 5A, the biocatalyst maintained high catalytic activity throughout five consecutive cycles, achieving levulinic acid conversion of approximately 97% without significant loss of activity. This result demonstrates excellent operational stability of the immobilized lipase under solvent-free conditions.

In general, repeated use of immobilized enzymes may lead to gradual activity loss due to thermal inactivation, enzyme leaching, structural denaturation, or degradation of the support material under mechanical stress [20]. However, the high stability observed in this study suggests that the reaction conditions—moderate temperature, effective water removal, relatively short reaction time, and compatible substrate properties minimized enzyme deactivation. The reagents and products can in principle transiently diffuse into and out of the porous structure of N435 during the reaction; hence a washing step between reuse cycles was introduced to minimize carryover of residual substrate and product trapped within the support. No visible fragmentation or physical deterioration of the immobilized biocatalyst was observed. The high level of preserved activity over consecutive reaction cycles (~ 97%) also signifies negligible enzyme leaching. The solvent-free systems such as the one used here are generally considered less prone to enzyme leaching than aqueous or highly polar reaction media, since there is no bulk aqueous phase to promote desorption of the physically adsorbed lipase from its support. Water generated as a by-product of esterification could, in principle, create locally hydrated microenvironments capable of promoting enzyme desorption; however, this water is continuously scavenged by the molecular sieves present in the reaction mixture.

From an industrial perspective, the demonstrated recyclability of the biocatalyst is particularly significant, as enzyme cost represents a major factor in enzymatic process economics. The ability to retain high catalytic performance over multiple cycles highlights the feasibility of applying this solvent-free esterification system for larger-scale production.

### Scaling up of active amyl levulinate and fusel levulinate production

**Fig. 6:**
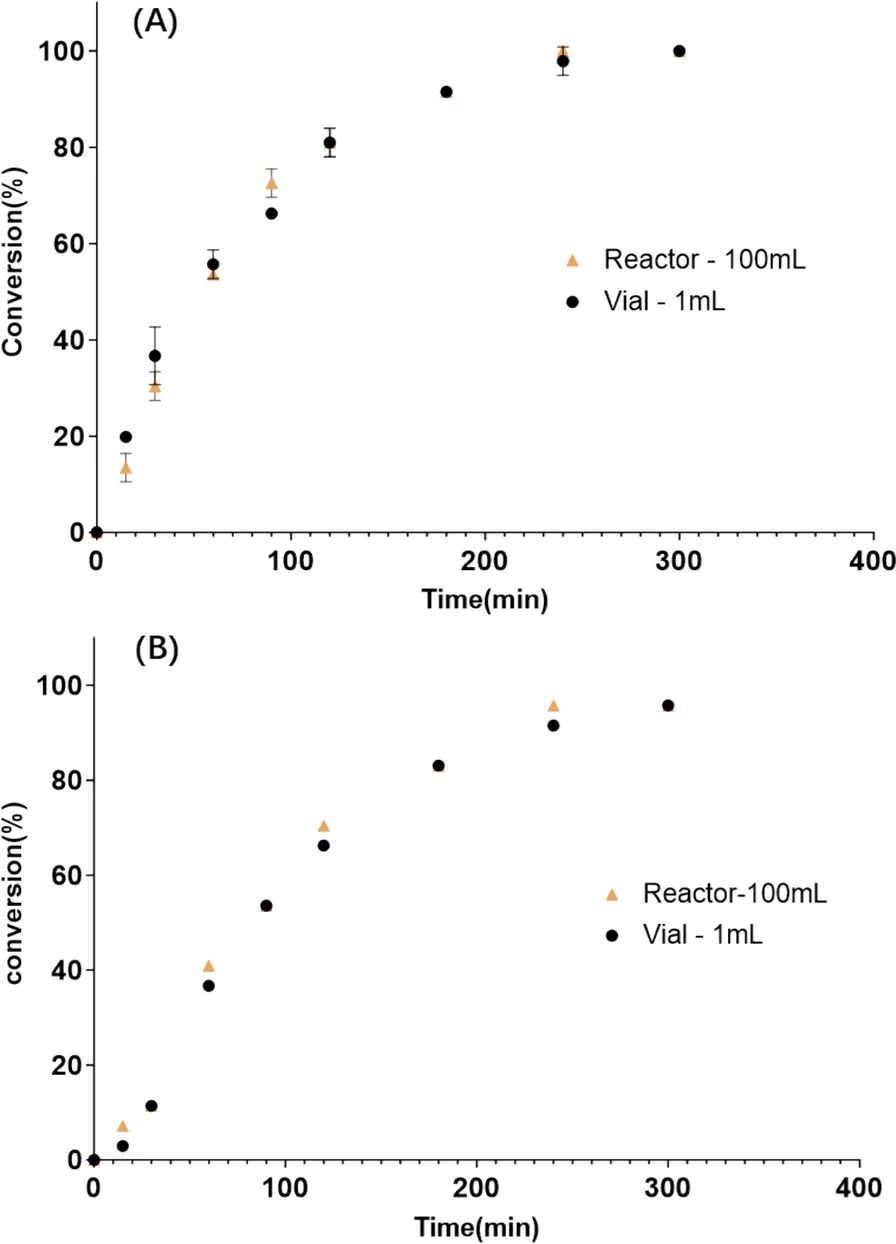
Production of **A** 3-methyl-1-butanol and **B** fusel levulinate at 1 mL and 100 mL scale, respectively. Reaction conditions: time 300 min, molar ratio of LA: 3-methyl-1-butanol (fusel oil) 1:10, enzyme amount 10% (w/w of acid), speed of agitation 600 rpm (in vial) and 400 rpm (in bioreactor), temperature 50 ℃, and molecular sieves 100% (w/w of acid)

To evaluate the scalability and kinetic consistency of the process, the reaction system was scaled up by approximately 100-fold, increasing the reaction volume from 1 to 2 mL (vial experiments) to ~100 mL in a Spinchem reactor with a working volume of 150 mL. Spinchem is a rotating bed reactor designed for heterogeneous (bio-)catalysis in which the solid phase is placed as a packed bed inside a rotating cylinder, creating a continuously circulating flow, hence enhancing mass transfer (https://spinchem.com/technology/). As shown in Fig. 6, esterification reactions using both 2-methyl-1-butanol and fusel alcohol exhibited highly comparable kinetic profiles between small-scale vial experiments and the scaled bioreactor system, demonstrating excellent scalability.

Although the vial-scale reactions displayed slightly faster conversion during the initial 30–60 min, the differences were minor and within experimental variation. After approximately 120 min, the reaction profiles became nearly identical, with both systems achieving near-complete conversion (> 98%) within 200–300 min. The small deviations observed during early reaction stages are likely attributed to scale-dependent physical factors rather than intrinsic changes in reaction kinetics. In larger reactor volumes, variations in mixing efficiency and substrate dispersion may temporarily reduce enzyme–substrate contact frequency. Additionally, differences in heating methods and the lower surface-area-to-volume ratio in the bioreactor can introduce greater thermal inertia, potentially delaying attainment of optimal reaction conditions. As the reaction progresses, these effects diminish, resulting in convergence of reaction rates and final conversion.

Overall, the results demonstrate that the esterification system maintains catalytic efficiency and kinetic behavior upon scale-up, highlighting its robustness and suitability for further process scale-up and intensification.

From a downstream processing perspective, the solvent-free process design simplifies product separation at larger scale. After completion of the reaction, the particulate immobilized biocatalyst and molecular sieves were readily removed from the liquid reaction mixture by filtration. Since alkyl levulinate esters are considerably less volatile than the parent fusel alcohols, the remaining excess alcohol could be selectively recovered from the ester product by rotary evaporation, as already demonstrated for the 100 mL scale-up reactions; the recovered alcohol can, in principle, be recycled directly into subsequent reaction batches. This contrasts with solvent-based systems, where an additional step is required to separate the ester product from both the excess alcohol and the organic co-solvent (e.g., MTBE or MIBK), and with homogeneous acid-catalyzed routes, which additionally require catalyst neutralization and generate salt waste that must be removed from the product stream. At larger manufacturing scales, distillation would likely replace rotary evaporation as the practical means of alcohol recovery, and evaluating the efficiency of this step, together with final product purity and biocatalyst performance over repeated cycles under continuous larger-scale operation, remains an important objective for future work.

Table 2 summarizes selected studies relevant to the present work, particularly those involving fusel alcohol-derived substrates, where isoamyl alcohol and active amyl alcohol are major components, and compares their outcomes with the results obtained in this study.

**Table 2:** Comparison of alkyl levulinate production by lipase-catalyzed esterification of levulinic acid with alcohols

Product	Immobilized lipase	Condition	Time	LA Conversion	References
3-Methyl-1-butyl levulinate	*C. antarctica* lipase B (SBA-15-APTS-GLU-Lip.)^a^	LA 0.01 mol (1.16g), 3-MB 0.02 mol (1.76 g), cat. 150 mg (13% LA), 50 °C, MTBE ^b^ 15 ml	2.5 h	94%	[41]
Butyl levulinate				85%	
3-Methyl-1-butyl levulinate	*C. antarctica* lipase B (immo-bead 150)	LA 7 mmol (812 mg, excess)^c^, 3-MB 4 mmol (352 mg), MTBE 2 mL, cat. 120 mg (34%, w/w IAA), 45 °C, 200 rpm	8 h	98% (IAA Conv.)^d^	[40] [42]
Butyl levulinate				99% (IAA Conv.)^d^	
3-Methyl-1-butyl levulinate	Imm-Lipase (Eversa® Transform 2.0) on PSty-DVB beads	1) 40 °C, cat. 20% (w/w reaction mixture mass), LA: 3-MB (molar ratio of 1:1.5), MIBK^e^ 500 mmol/L 2) 40 °C, cat. 10% (w/w reaction mixture mass), LA:3-MB (molar ratio of 1:1), Solvent-free	12 h	65%	[10]
			24 h	app. 8%	
3-Methyl-1-butyl levulinate 2-Methyl-1-butyl levulinate 2-Butyl levulinate Fusel alcohols	*C. antarctica* lipase B, Novozym 435	50 °C, cat. 10% (w/w LA), LA:alcohols (1:5, w/w), 100% molecular sieves, Solvent-free	5 h 5 h 5 h 5 h	> 99% > 99% > 70% > 99%	This study

a. SBA-15-APTS-GLU-Lip; Immobilized *C. antarctica* lipase B on the surface functionalized SBA-15

b. MTBE; Methyl tert-butyl ether

c. LA was used in excess of IAA, while in all other studies, IAA was used in excess of LA

d. Calculated by conversion of IAA, instead of LA

e. MIBK; Methyl isobutyl ketone

As illustrated in Table 2, esterification performance is highly dependent on both enzyme selection and reaction environment. High yield (94–98%) of isoamyl levulinate from levulinic acid and isoamyl alcohol was reported using CALB immobilized to different matrices in methyl tert-butyl ether (MTBE) [41, 50]. This would require additional downstream processing to remove the solvent and residual alcohol, thereby increasing operational complexity and cost. Notably, conversion was calculated based on alcohol consumption rather than levulinic acid conversion, despite levulinic acid being present in excess. This complicates direct comparison with other studies and may overestimate practical substrate utilization. In another study, esterification of isoamyl alcohol with levulinic acid performed using Eversa® Transform 2.0 (a lipase marketed for biodiesel production) immobilized on poly(styrene–divinylbenzene) beads in methyl isobutyl ketone (MIBK) led to a maximum levulinic acid conversion of 65% after 12 h [10]. However, when the reaction was conducted under solvent-free conditions, conversion decreased significantly to approximately 8%, highlighting the strong dependence of enzymatic activity on reaction medium. The biocatalyst retained around 30% of its initial activity after five consecutive esterification batches under optimal experimental conditions [10].

Compared with previously reported systems, the process developed in this study demonstrates competitive performance, particularly in achieving efficient esterification under solvent-free conditions while maintaining operational simplicity. The process shows promise for further scale-up for utilizing crude fusel alcohol mixtures for levulinate ester production and evaluation of the product as renewable fuel oxygenate.

## Conclusion

This study demonstrates an efficient solvent-free enzymatic approach for the synthesis of alkyl levulinates through lipase-catalyzed esterification of levulinic acid with fusel oil from bioethanol production. Systematic optimization of reaction parameters enabled nearly complete conversion of levulinic acid (~ 99%) within 5 h under mild reaction conditions. The optimized system exhibited high catalytic performance, operational stability, along with preliminary scalability, as confirmed by successful scale-up experiments in a lab-scale bioreactor. The solvent-free process eliminates the need for additional organic solvents, simplifying downstream processing and improving overall process sustainability. Excess alcohol can be easily recovered and recycled, while enabling the formation of a high-purity product.

Molecular docking analysis of the primary alcohols present in fusel oil and a secondary alcohol (2-butanol) further provided mechanistic insight into substrate-dependent reaction rates, highlighting the combined influence of binding strength, conformational compatibility, and the ability of substrates to adopt catalytically productive geometries required for the deacylation step.

Further studies on developing other process operation modes such as continuous-flow or recycling reactor systems will be important to validate long-term operational stability, enhance process efficiency, and support future scale-up. Overall, this work presents a straightforward and sustainable strategy for converting renewable biomass-derived feedstocks into valuable alkyl levulinates with potential applications as green solvents, specialty chemicals, and renewable fuel oxygenates, thereby contributing to circular bioeconomy concepts. By displacing petroleum-derived ethers and esters currently used as fuel oxygenates with bio-based alkyl levulinates, this approach can help reduce fossil carbon input into the fuel additive supply chain, consistent with circular bioeconomy principles that prioritize biogenic and waste-derived carbon over virgin fossil resources [1, 2, 4, 12].

## Supplementary Information

Supplementary Material 1.

## Data Availability

No datasets were generated or analyzed during the current study.
