# Supplementary material for: Biocatalytic upgrading of fusel oil from bioethanol production to levulinate esters as renewable fuel oxygenates
Source: Biotechnol Biofuels Bioprod. 2026 Sep 8;19:68. doi: 10.1186/s13068-026-02816-9 (PMC13551973; doi:10.1186/s13068-026-02816-9)
Supplement: Supplementary file 1 [file 13068_2026_2816_MOESM1_ESM.pdf]

Supplementary Information

Biocatalytic Upgrading of Fusel Oil from Bioethanol Production To Levulinate Esters as Renewable Fuel Oxygenates

*Yuchen Luo, Mohamed Ismail, Rajni Hatti-Kaul and Sang-Hyun Pyo **

Biotechnology & Applied Microbiology, Department of Process & Life Science Engineering, Faculty of Engineering, Lund University, SE-22100 Lund, Sweden

* Corresponding author
E-mail address: sang_hyun.pyo@ple.lth.se (S.-H. Pyo)

Table of Contents

Figure S1. The mechanism of lipase-catalyzed levulinate synthesis 3

Figure S2. (A) 1H- and (B) ^{13}C NMR spectra of the product obtained after 4 h of esterification between levulinic acid and 2-methyl-1-butanol, following removal of excess alcohol by simple evaporation. 4

Figure S3. FT-IR spectra of the reaction mixture at (A) the initial stage (0 min) and (B) the final stage (300 min) following removal of residual alcohol. 5

Figure S4. (A) ^{1}H-NMR, (B) ^{13}C NMR and FT-IR spectra of the product obtained after 4 h of esterification between levulinic acid and fusel oil, following removal of excess alcohol by simple evaporation. 6

Figure S5. (A) ^{1}H-NMR, (B) ^{13}C NMR and FT-IR spectra of levulinic acid............................. 7

Figure S6. ^{1}H-NMR spectra of (A) 2-methyl-1-butanol, (B) 3-methyl-1-butanol, and (C) fusel oil. 8-9

Figure S7. Figure S7. GC-MS chromatograms of (A) 1-butanol (RT2.898), (B) 3-methyl-1-butanol (RT3.873), (C) 2-Methyl-1-butanol (RT3.926), (D) Fusel oil (RT3.857, RT3.900), (E) Fusel oiil-pretreated (RT3.8533, RT3.883), and (F) Fusel levulinate (RT9.764/9.78) 10

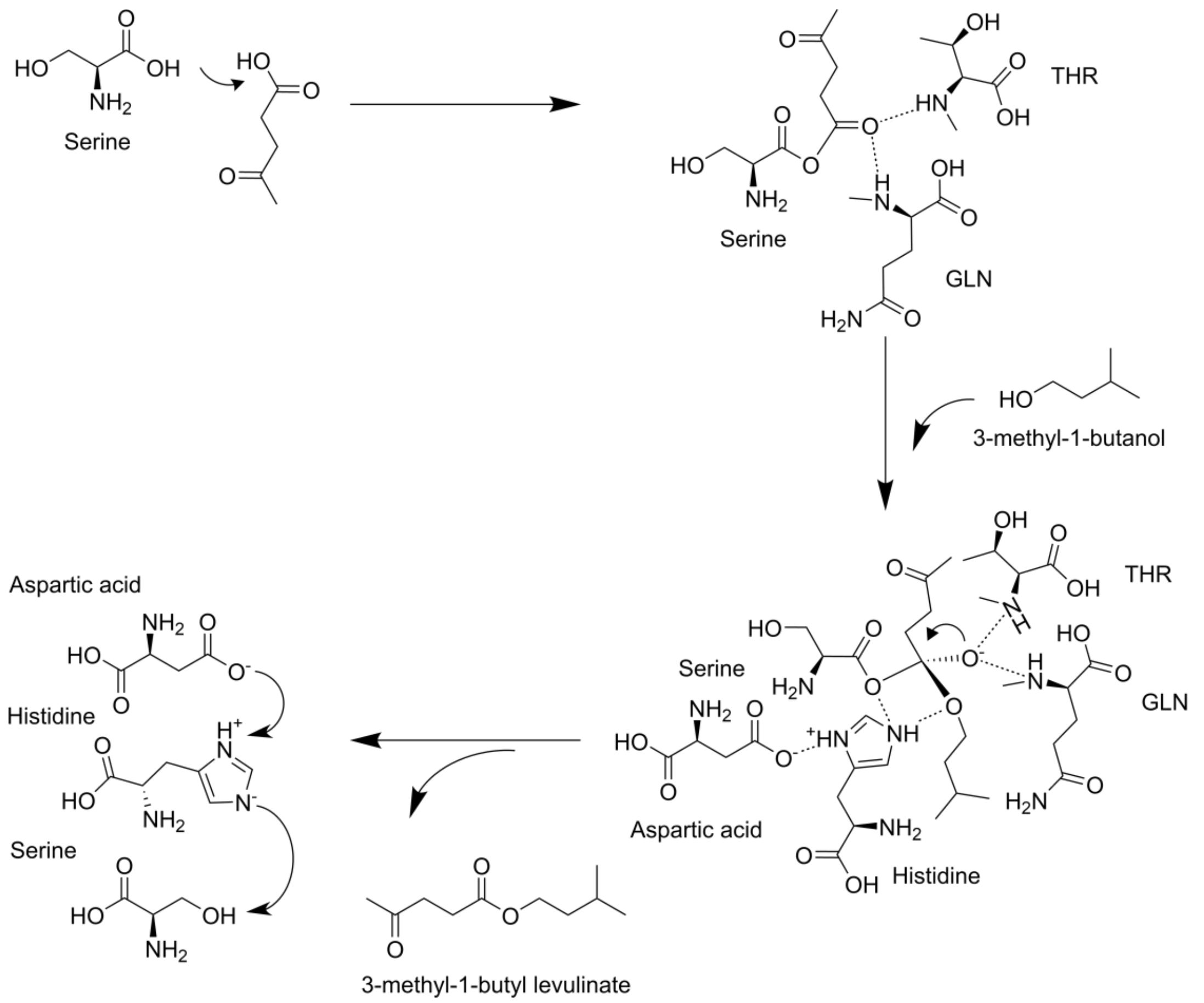

Figure S1. The mechanism of lipase-catalyzed levulinate synthesis.

(A)

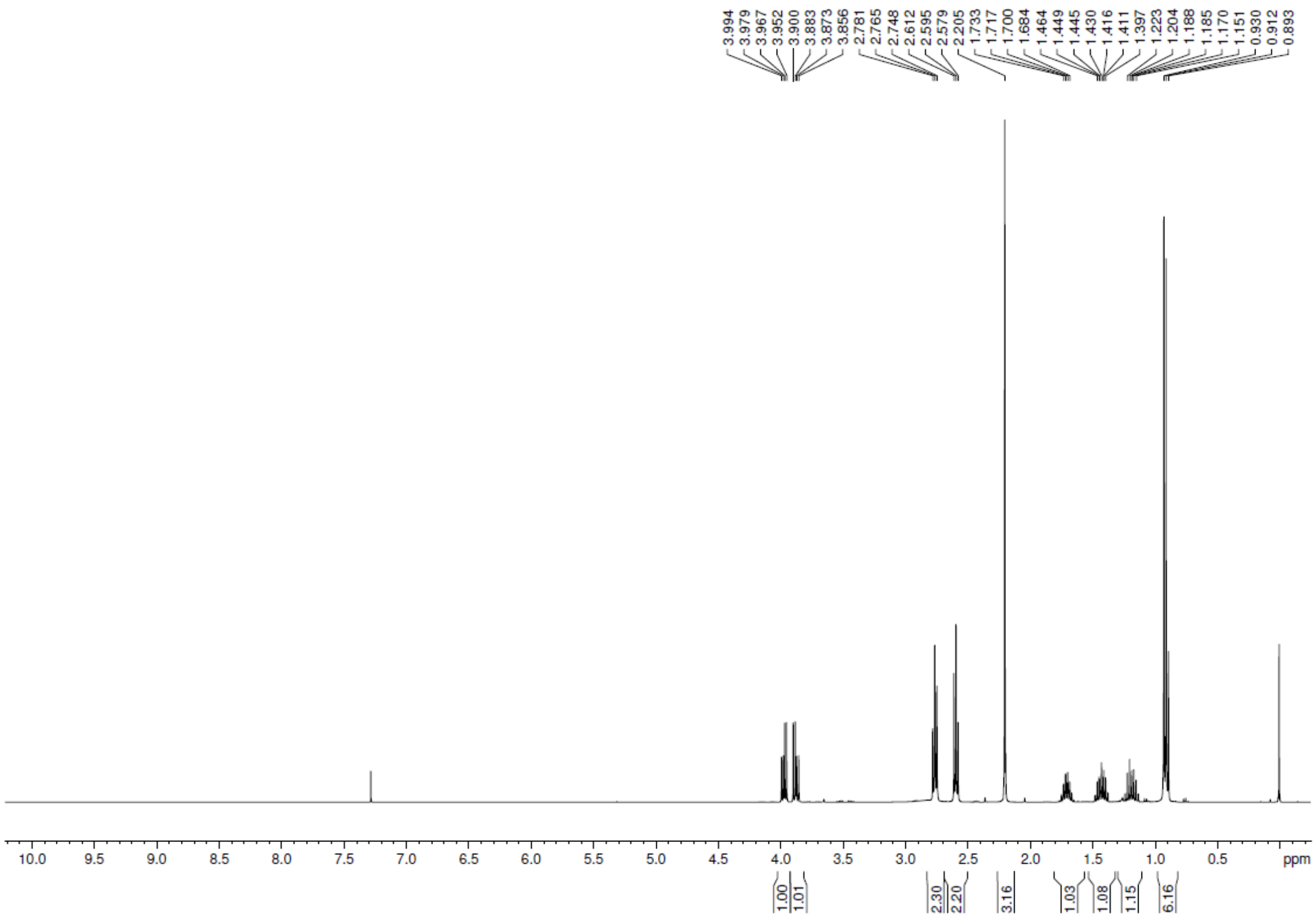

(B)

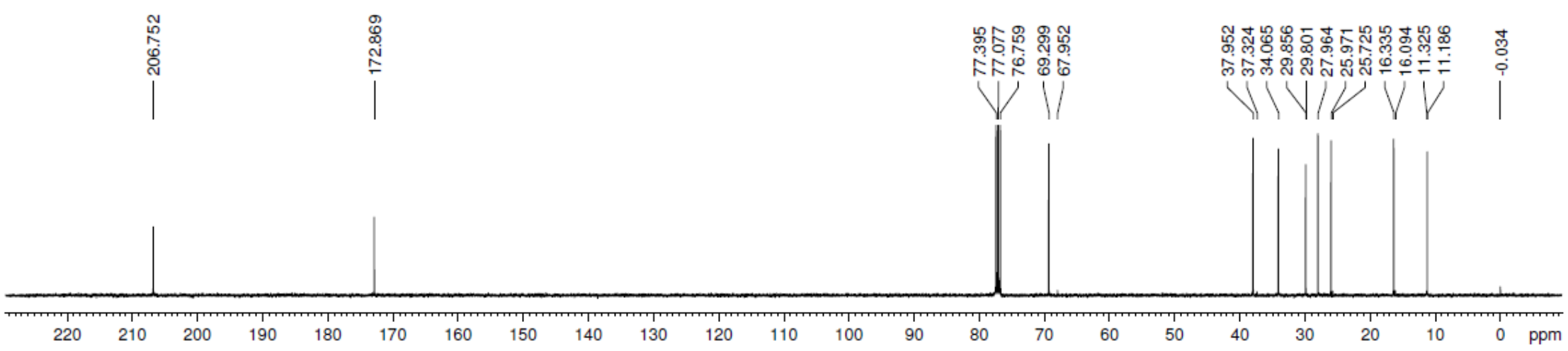

(C)

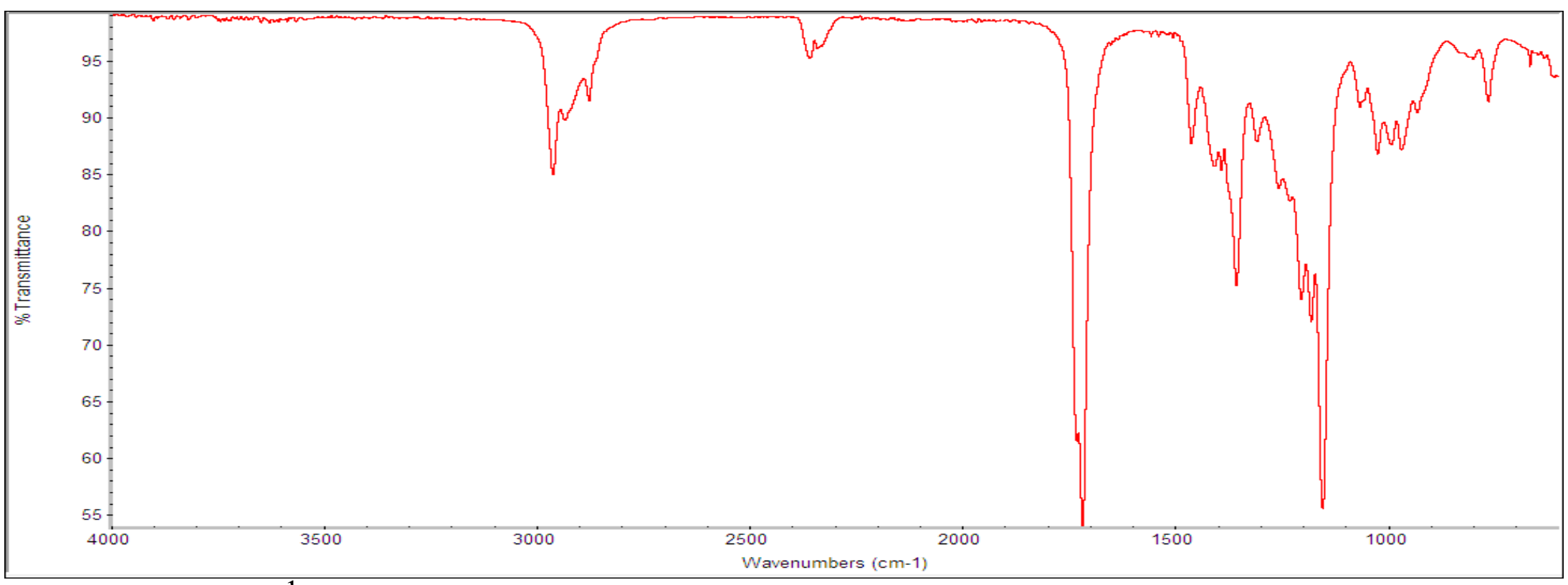

Figure S2. (A) ^{1}H-NMR, (B) ^{13}C NMR and FT-IR spectra of the product obtained after 4 h of esterification between levulinic acid and 2-methyl-1-butanol, following removal of excess alcohol by simple evaporation.

(A)

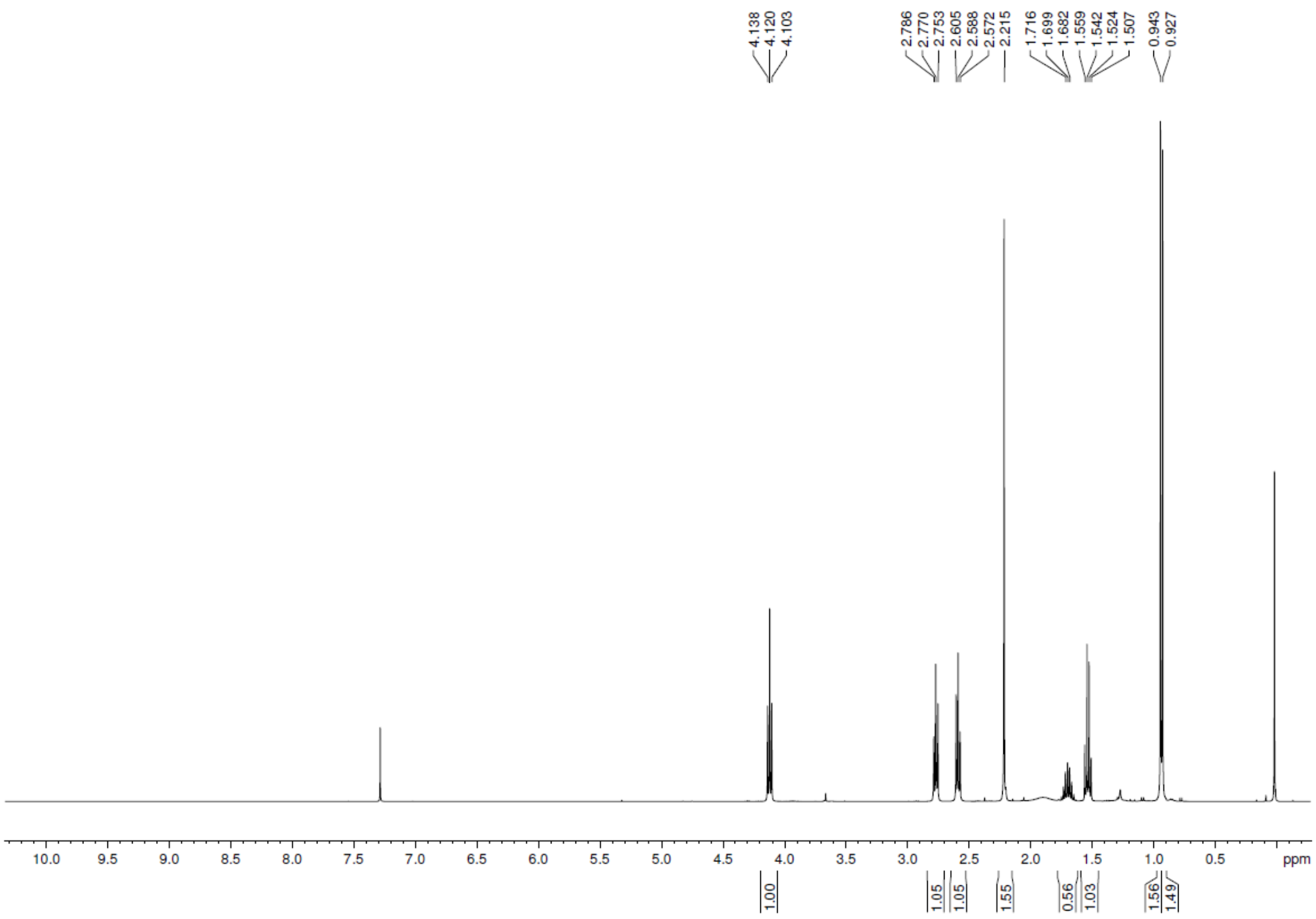

(B)

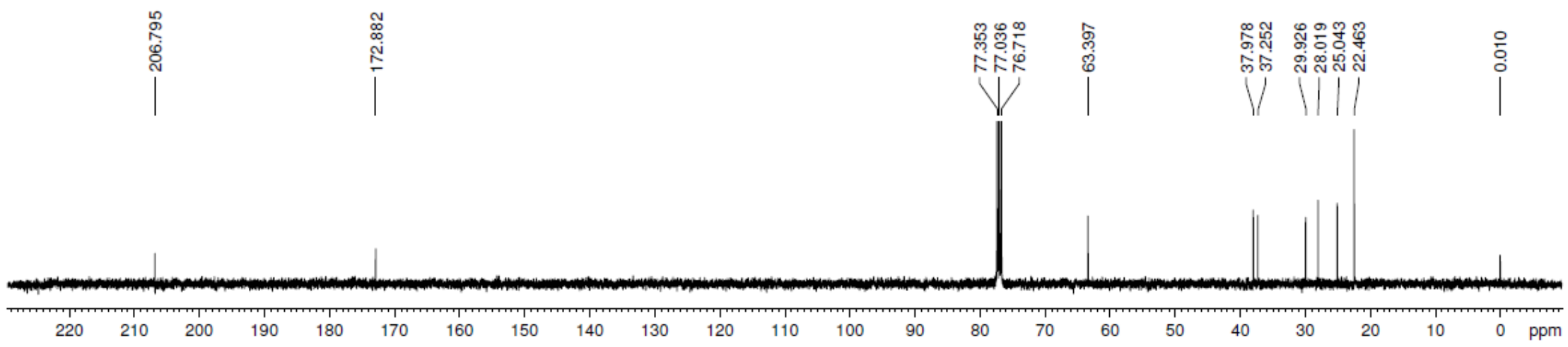

(C)

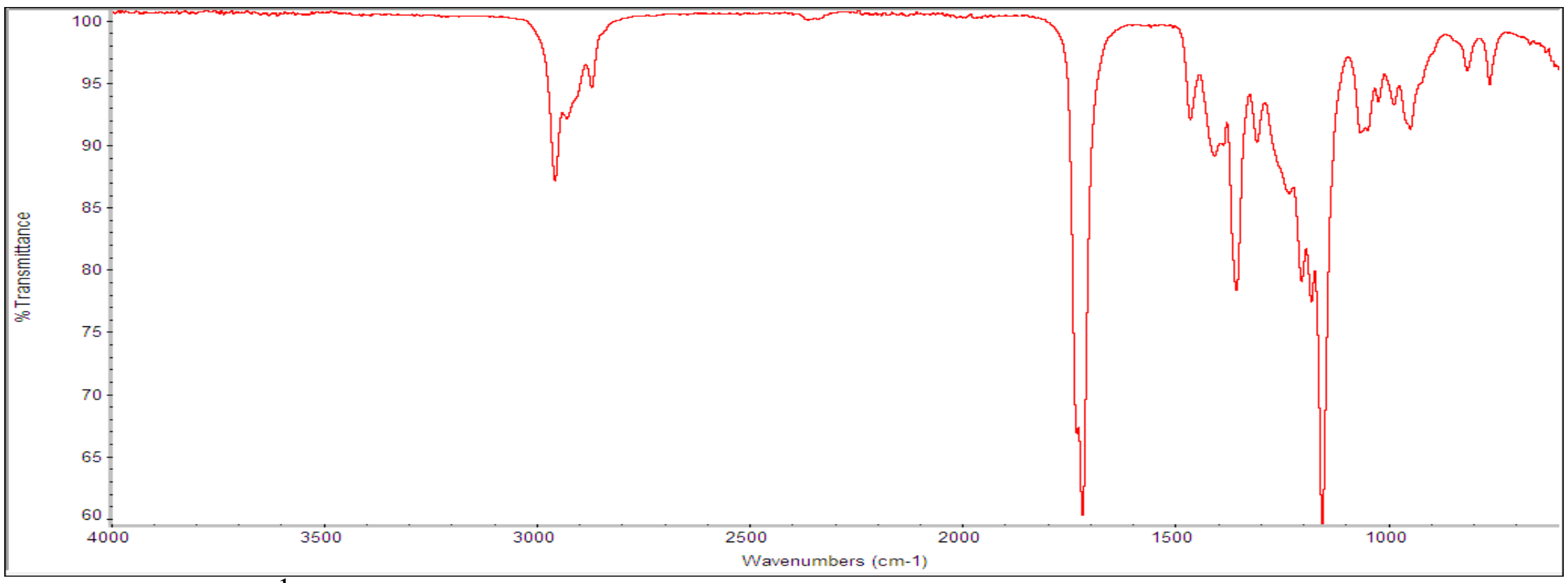

Figure S3. (A) ^{1}H-NMR, (B) ^{13}C NMR and FT-IR spectra of the product obtained after 4 h of esterification between levulinic acid and 3-methyl-1-butanol, following removal of excess alcohol by simple evaporation.

(A)

(B)

(C)

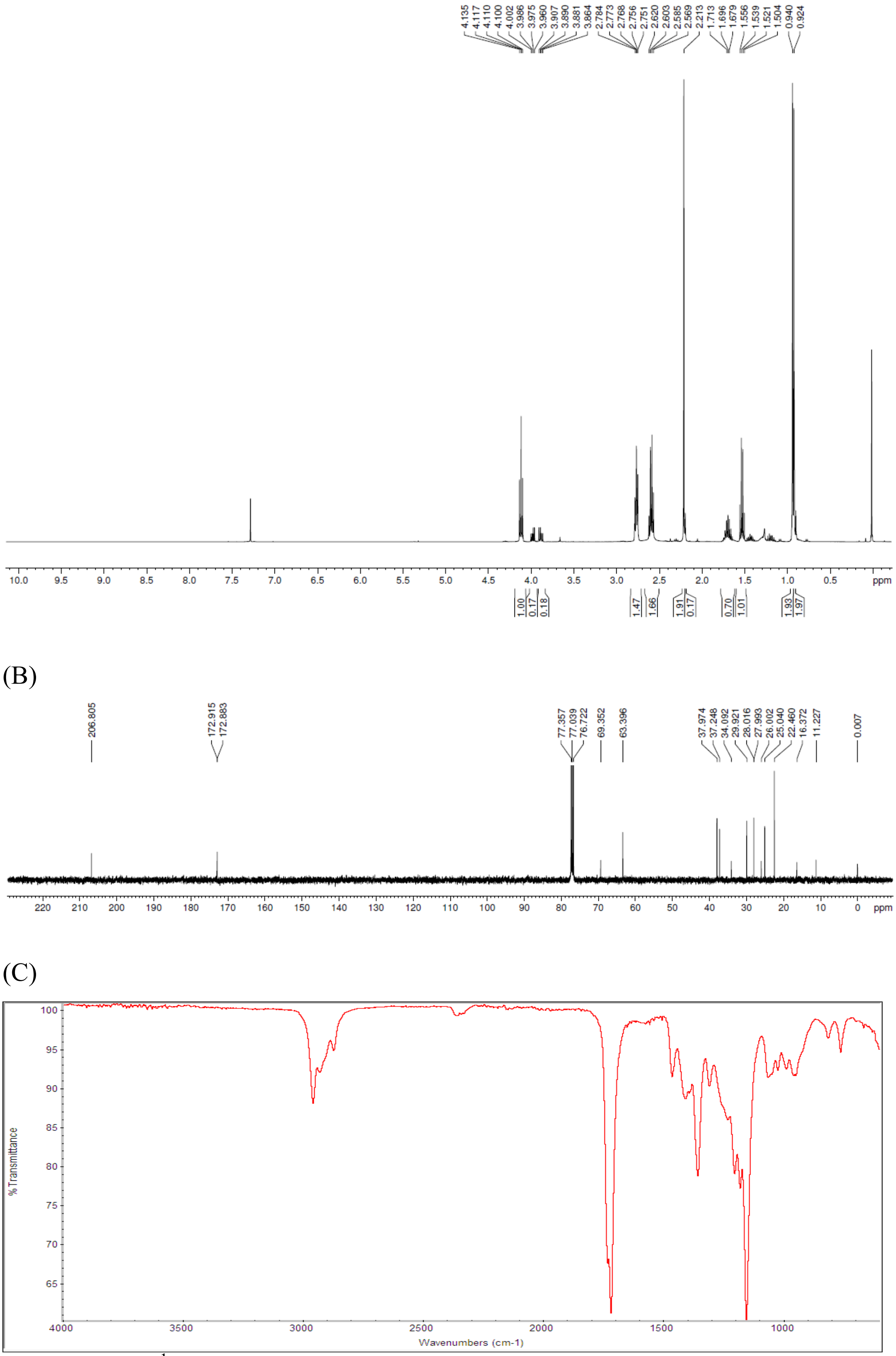

Figure S4. (A) ^{1}H-NMR, (B) ^{13}C NMR and FT-IR spectra of the product obtained after 4 h of esterification between levulinic acid and fusel oil, following removal of excess alcohol by simple evaporation.

(A)

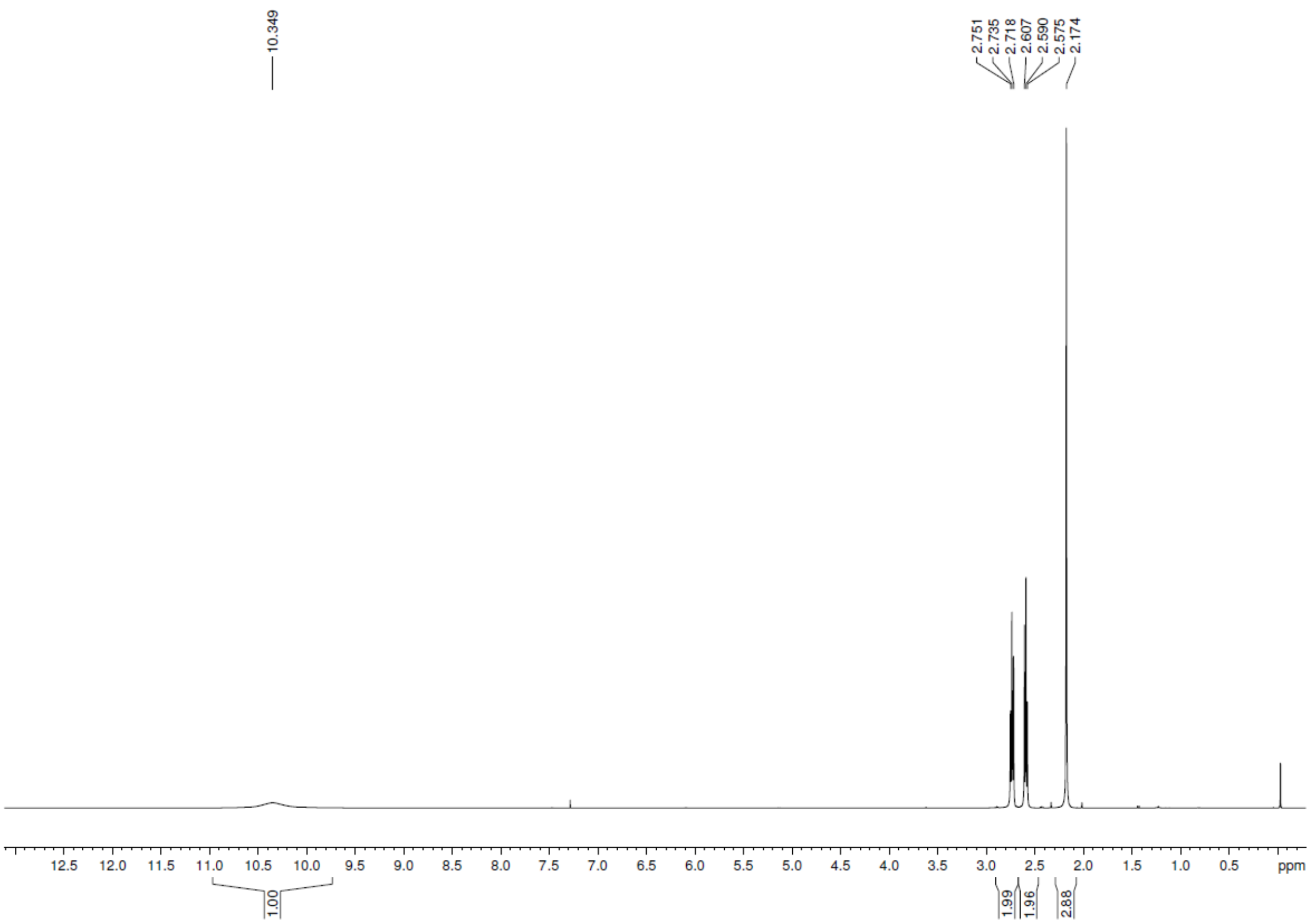

(B)

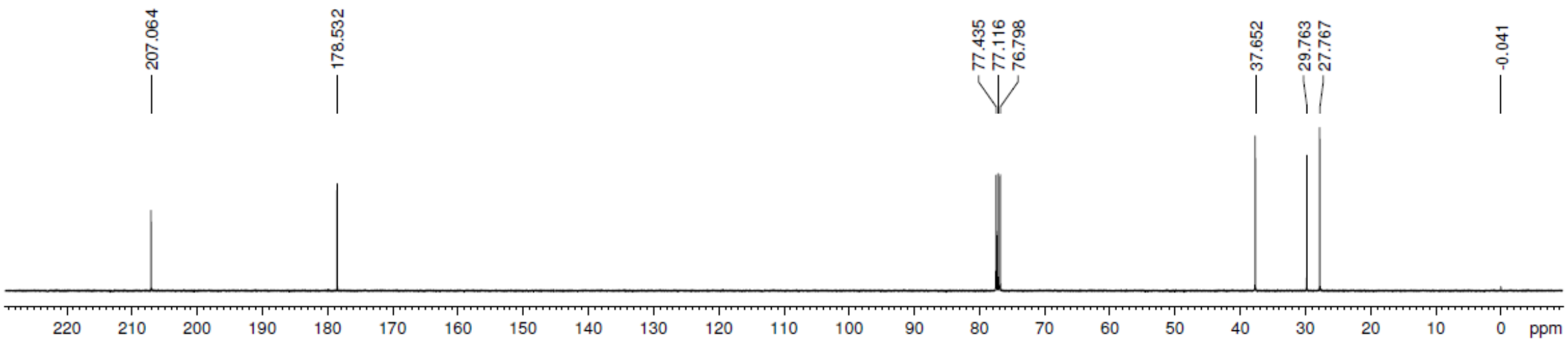

(C)

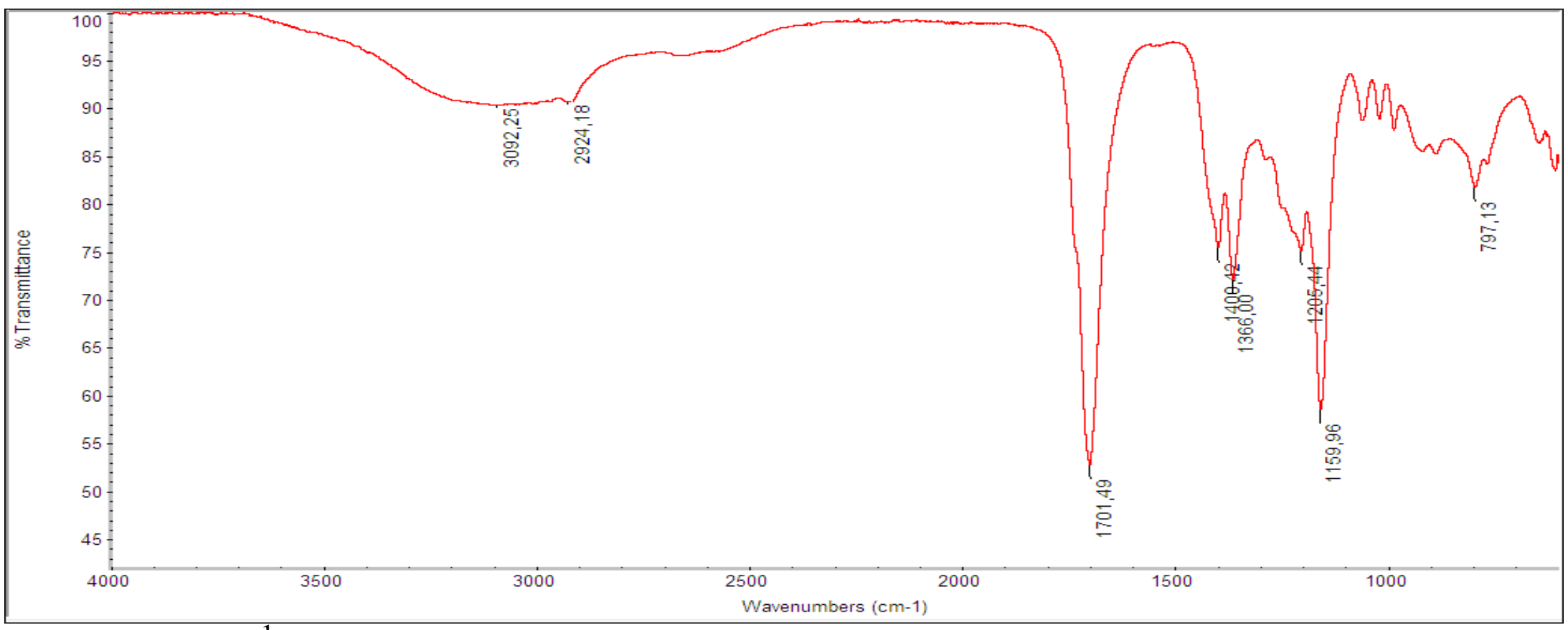

Figure S5. (A) ^{1}H-NMR, (B) ^{13}C NMR and (C) FT-IR spectra of levulinic acid

(A)

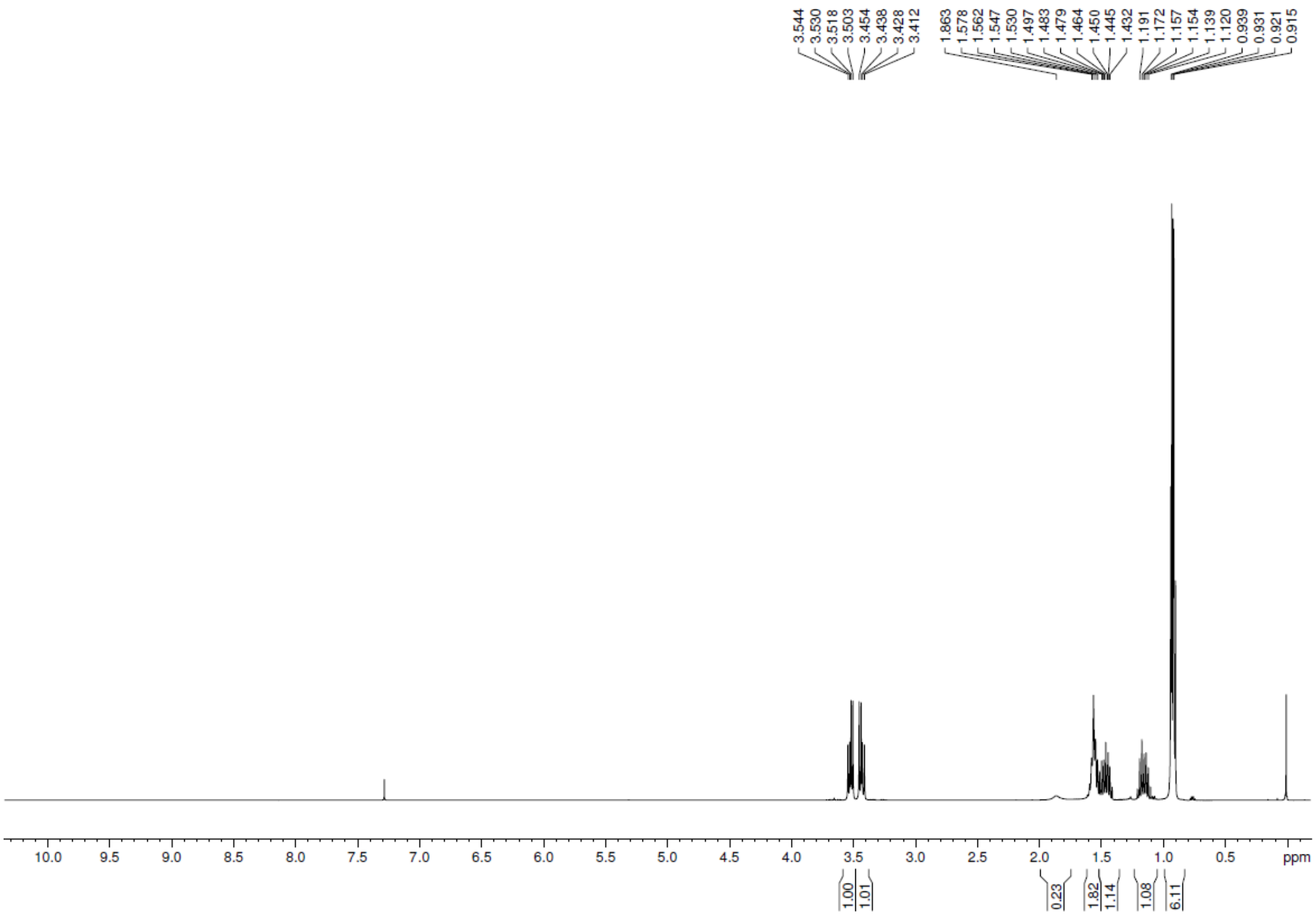
3.544
3.530
3.518
3.503
3.454
3.438
3.428
3.412
1.863
1.578
1.562
1.547
1.530
1.497
1.483
1.479
1.464
1.450
1.445
1.432
1.191
1.172
1.157
1.154
1.139
1.120
0.939
0.931
0.921
0.915
10.0 9.5 9.0 8.5 8.0 7.5 7.0 6.5 6.0 5.5 5.0 4.5 4.0 3.5 3.0 2.5 2.0 1.5 1.0 0.5 ppm
1.00
1.01
0.23
1.82
1.14
1.08
6.11

(B)

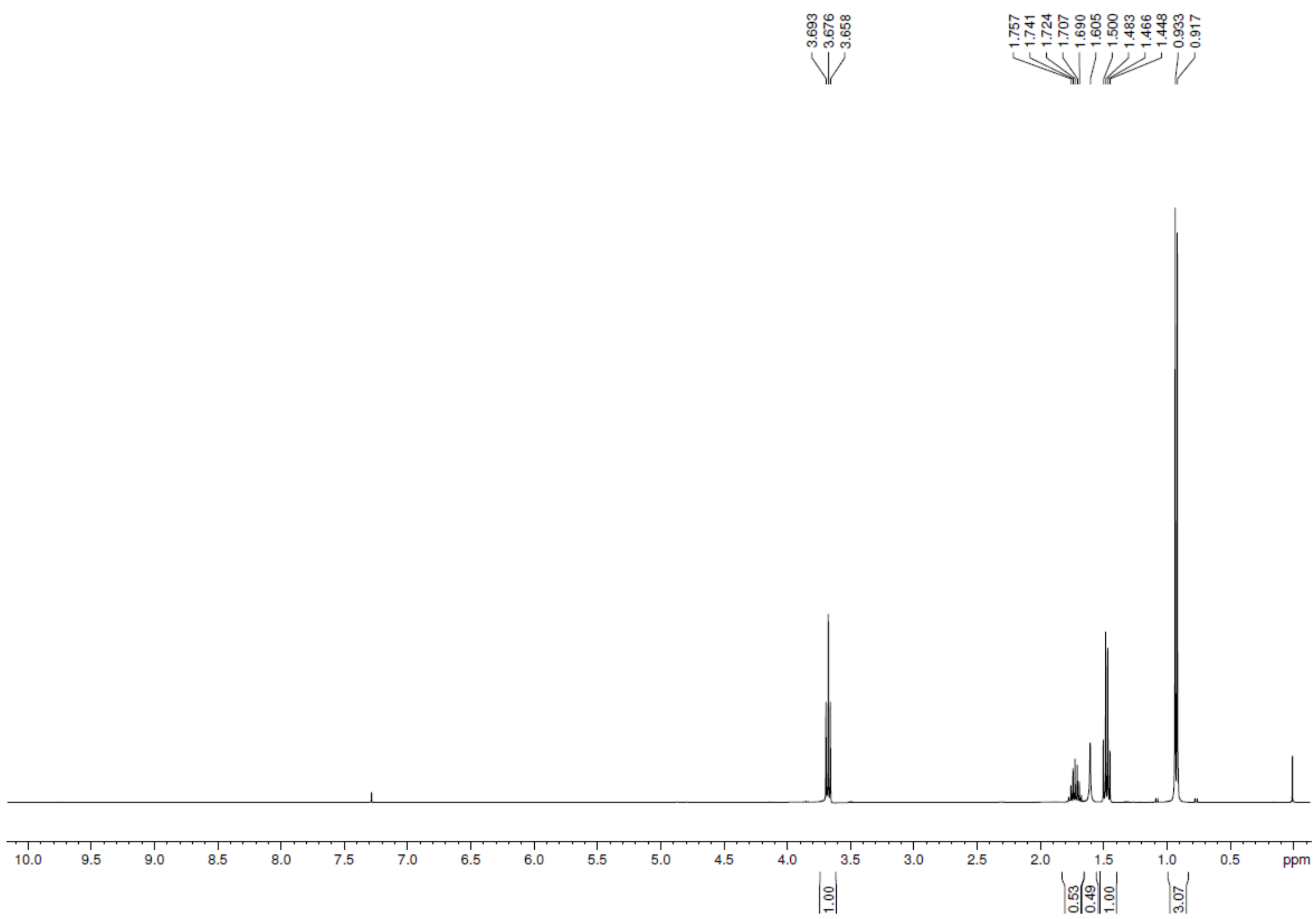
3.693
3.676
3.658
1.757
1.741
1.724
1.707
1.690
1.605
1.500
1.483
1.466
1.448
0.933
0.917
10.0 9.5 9.0 8.5 8.0 7.5 7.0 6.5 6.0 5.5 5.0 4.5 4.0 3.5 3.0 2.5 2.0 1.5 1.0 0.5 ppm
1.00
0.53
0.49
1.00
3.07

(C)

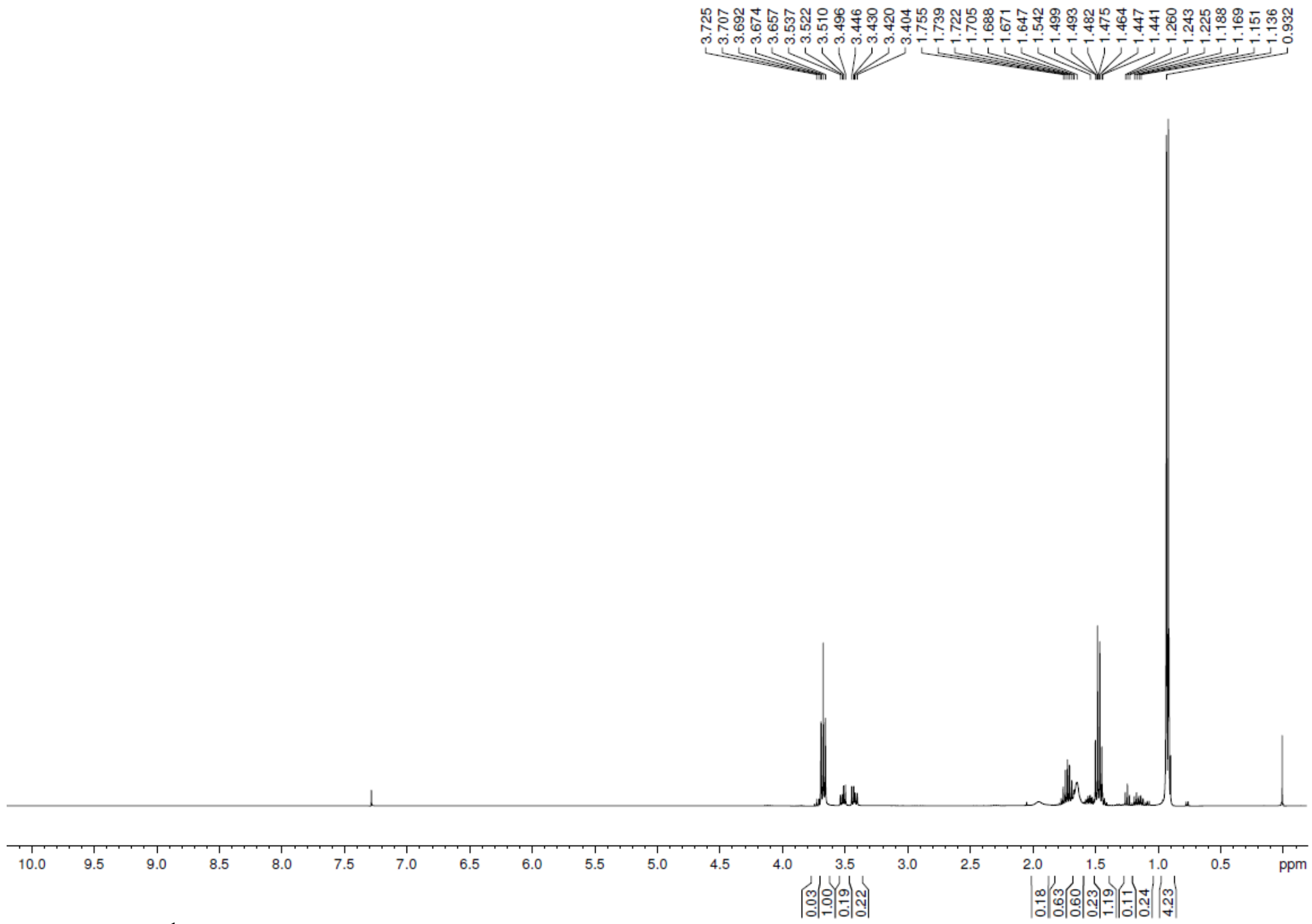

Figure S6. ^{1}H-NMR spectra of (A) 2-methyl-1-butanol, (B) 3-methyl-1-butanol, and (C) fusel oil.

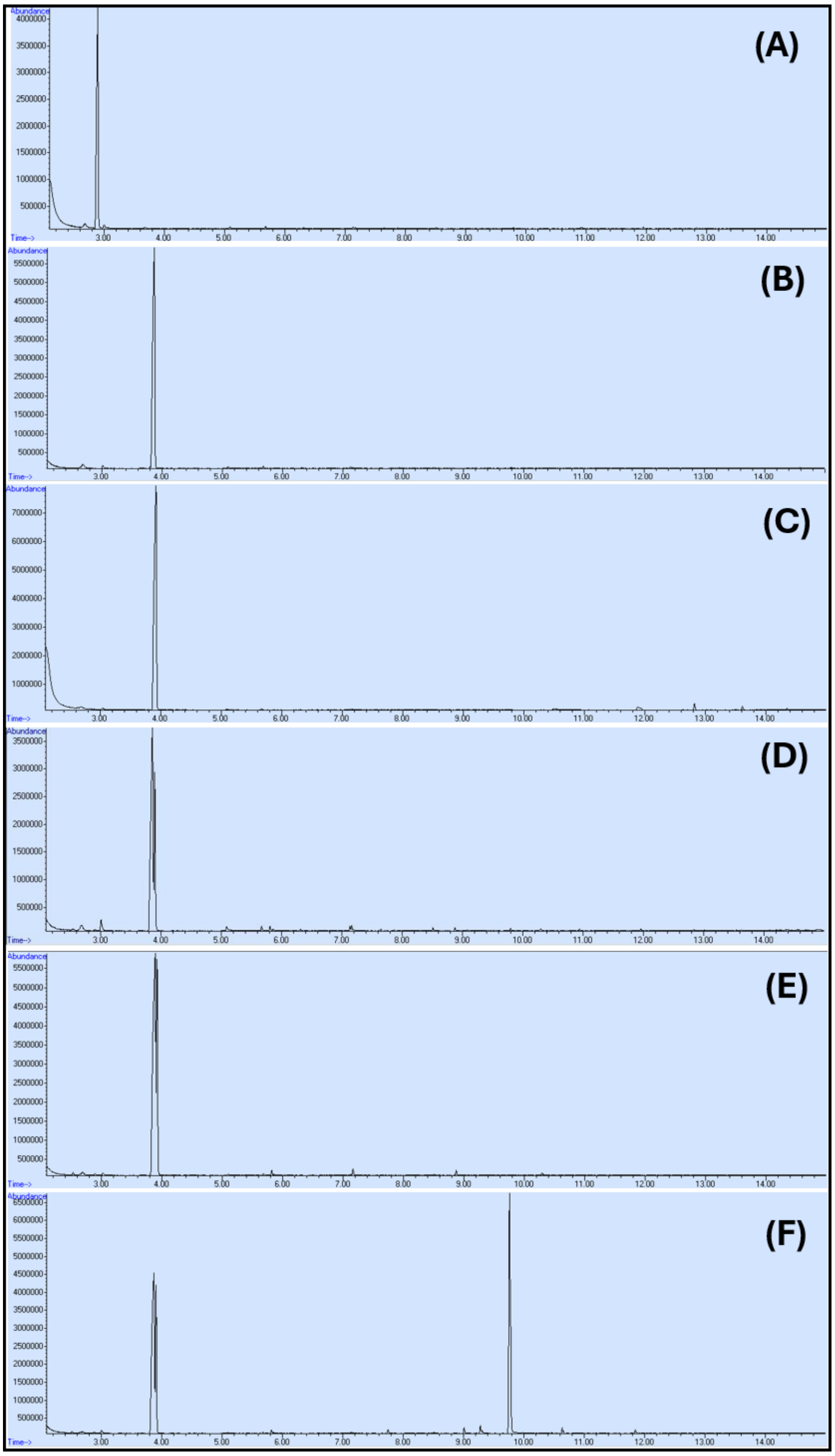

Figure S7. GC-MS chromatograms of (A) 1-butanol (RT2.898), (B) 3-methyl-1-butanol (RT3.873), (C) 2-Methyl-1-butanol (RT3.926), (D) Fusel oil (RT3.857, RT3.900), (E) Fusel oil-pretreated (RT3.8533, RT3.883), and (F) Fusel levulinate in fusel reaction solution (RT9.764/9.78).
